# Risk factors for short-term all-cause mortality in patients with end stage renal disease: a scoping review

**DOI:** 10.1186/s12882-024-03503-3

**Published:** 2024-02-27

**Authors:** Wanfen Yip, Sheryl Hui Xian Ng, Palvinder Kaur, Pradeep Paul George, Jennifer Huey Chen Guan, Guozhang Lee, Timothy Jee Kam Koh, Woan Shin Tan, Allyn Yin Mei Hum

**Affiliations:** 1 Health Services & Outcomes Research, National Healthcare Group, Singapore, Singapore; 2 Department of Palliative Medicine, Tan Tock Seng Hospital, Singapore, Singapore; 3 Department of Internal Medicine, Singapore General Hospital, Singapore, Singapore; 4 Department of Renal Medicine, Tan Tock Seng Hospital, Singapore, Singapore; 5 Geriatric Education and Research Institute, Singapore, Singapore; 6 The Palliative Care Centre for Excellence in Research and Education, Dover Park Hospice, Singapore, Singapore

**Keywords:** End-stage renal disease, Renal supportive care, Palliative care, Risk factors for short-term mortality, Risk factors for all-cause mortality

## Abstract

**Objectives:**

There is a lack of prognostic information to guide the prediction of short-term all-cause mortality in patients with end-stage renal disease (ESRD). The aim was to review the risk factors that influenced the risk of short-term all-cause mortality in patients with ESRD.

**Methods:**

MEDLINE, Embase, PubMed, CINAHL, the Cochrane Library and Web of Science databases were searched for articles published between 2000 and 2020. Articles describing risk factors predicting short-term mortality (≤ 3 years) in patients with ESRD were included. Four reviewers independently performed title, abstract, full text screening and data extraction. Assessment of risk of bias was assessed using the Quality In Prognosis Studies (QUIPS) tool checklist.

**Results:**

20,840 articles were identified and 113 papers were included for this review. Of the 113 papers, 6.2% included only peritoneal dialysis (PD) patients, 67.3% included only hemodialysis (HD) patients, 20.4% included both PD and HD patients, with the remaining papers featuring patients on conservative management or awaiting renal transplant. Risk factors were categorised into 13 domains: 1)demographics/ lifestyle, 2) comorbidities 3)intradialytic blood pressure, 4)biomarkers, 5)cardiovascular measurements, 6)frailty status, 7)medications, 8)treatment related indicators, 9)renal related parameters, 10)health status, 11)cause of ESRD, 12)access to healthcare care/ information and, 13)proxy measures for poor health. C-reactive protein(CRP), age, and functional status were observed to have higher percentage of instances of being significantly associated with all-cause mortality.

**Conclusion:**

Commonly examined risk factors observed from this review may be used to build a general prognostic model for patients with ESRD, with specific treatment related risk factors added on to enhance the accuracy of the models.

**Supplementary Information:**

The online version contains supplementary material available at 10.1186/s12882-024-03503-3.

## Introduction

The incidence of end-stage renal disease (ESRD) will grow with an increasingly aged population suffering with multimorbidity [1, 2]. Between 1990 and 2017, the global prevalence of chronic kidney disease (CKD) increased by 29.3% to affect 700 million individuals [3, 4]. The age-standardised incidence of ESRD treated with dialysis increased by 43% [3, 4], whilst deaths due to CKD increased by 41.5% between 1990 and 2017 [3, 4]. ESRD is a costly and disabling condition with a high mortality rate [3]. Patients with ESRD have a high symptom burden, and consequently a poor quality of life [5, 6]. Given the impact of symptoms experienced by ESRD patients, renal supportive care is an important resource in the holistic management of the patients’ evolving needs throughout their disease trajectory [5, 7]. 

Despite the importance and potential benefits, renal supportive care is underutilised [7]. In the United States, dialysis patients accounted for only 3% of those admitted to hospice with non-cancer diagnosis, and a small minority of dialysis patients who died had ever received hospice or palliative care services [7]. In addition, patients are often referred late in their disease trajectory, resulting in presentations dominated by complex care needs and continued reliance on the acute care system [7]. Current barriers to renal supportive care include a lack of understanding of benefits involved, inadequate palliative services in a straitened health care workforce and lack of standardised referral frameworks [8–10]. A lack of understanding of the benefits also contributed to patients’ negative perceptions and rejection of renal supportive care even when referred [8]. In the United States, 24% of patients who discontinued dialysis rejected hospice care, suggesting a lack of understanding of the benefit to symptom support at the end of life [11]. Inadequate palliative care services also meant that there were insufficient resources to provide care for patients [10]. In the United States, nearly one third of hospitals (702 out of 2293) with more than 50 beds do not have palliative care services [12]. Further, in a survey conducted among American and Canadian clinicians, nephrologists tend to be poorly trained in palliative care and remain uncomfortable in this aspect of clinical care. Together, these barriers result in uneven access to renal supportive care services and underdeveloped models of care [7, 13].

The lack of standardised frameworks affect timely referrals to renal supportive care to meet the needs of these patients [9]. A standardised referral framework based on needs assessment is preferred to identify patients for renal supportive care to avoid the pursuit of aggressive treatment options when the disease trajectory is one of decline. However, such a framework is currently lacking for ESRD, contributing to patients’ pursuit of aggressive treatment options without a holistic understanding of their health condition and limitations of treatment [14]. To facilitate identification of patients in a timely manner, in alignment with institutional resource capacity to provide care, prognostication models can help identify patients for palliative needs assessments and subsequent referral. The Kidney Disease Improving Global Outcomes (KDIGO) and the American Society of Nephrology ‘Choosing Wisely’ campaign emphasise that individualised prognostic information should be utilised to help patients and families make informed decisions regarding choice of treatment based on disease trajectory to avoid decisional regret [15–18]. Prognostic information will better prepare clinicians for realistic discussions about medical treatments and limitations with patients and their family, with a view to offering continued support with renal supportive care [19, 20].

The average life expectancy of ESRD patients on renal replacement therapy or conservative treatment is 2–10 years [21, 22]. Therefore, an individualised prognostic framework based on 3-year mortality will support decision making for patients and families by providing valuable information on treatment options and likely outcomes [23]. Additionally, it will aid clinicians in identifying patients who require renal supportive care, enabling clinicians to share evidence-based prognostic information, prognosis and goals of care with patients [21, 23, 24]. Recent reviews on prognostic factors have a wide mortality risk period range (e.g., 1 to 10 years) [25–29], and tend to be focused on specific populations (e.g., elderly patients on haemodialysis or patients on peritoneal dialysis) [25–29], limiting the generalisability and clinical utility of the findings. For example, prognostic information specific to the population of elderly patients on haemodialysis may not be applicable to patients on conservative treatment or peritoneal dialysis. Critically, there are a lack of prognostic models for patients on conservative management or at the time of treatment.

Hence, the aim was to review risk factors that influence 3-year all-cause mortality in patients with ESRD. Findings from this study will provide important information to build prognostic tools to support early identification of patients with ESRD who would benefit from integrated renal supportive care interventions.

## Methods

A scoping review was systematically conducted by adopting a five-stage methodological framework proposed by Arksey and O’Malley [30, 31]. Summarisation and reporting of the evidence were guided by the Preferred Reporting Items for Systematic Reviews (PRISMA) Extension for Scoping Reviews checklist and published guidelines for prognostic factor studies [32, 33].

### Search strategy

Any study that examined risk factors for short-term all-cause mortality (≤ 3 years) in patients with ESRD was eligible for inclusion in the current review. MEDLINE, Excerpta Medical database (EMBASE), PubMed, Cumulative Index to Nursing and Allied Health Literature (CINAHL), Cochrane Library and Web of Science electronic databases were searched for articles published from 2000 up to 11 November 2020. The search strategies were co-drafted with a librarian, and further edited through team discussions with inputs from palliative care physicians and nephrologists (Supplementary Tables 1–2).

### Eligibility criteria

We included original research articles and observational studies which described factors that predicted short-term all-cause mortality in patients with ESRD or conducted validation of existing models. A study was eligible for inclusion if the following criteria was met: (1) the study included patients with ESRD or on renal replacement therapy or conservative treatment aged 18 years and above; (2) papers which either reported multivariable analysis or conducted validation of existing models (Supplementary Table 1).

We excluded papers not specific to patients with ESRD, reporting competing risk analyses (Supplementary Table 1). Non-English language articles, letters, editorials, narrative reviews, commentaries, and care reports were excluded.

Titles and abstracts screening were independently undertaken by four different reviewers. Any inconsistencies between two reviewers were settled through discussions amongst the four authors. Full-text screening was undertaken by four different authors. Disagreements between the two authors were resolved by a third reviewer.

### Data extraction

Data extraction was performed by four reviewers. Disagreements were resolved through discussions with a third reviewer. Information regarding study design, sample size, missing data, statistical methodology, outcome information, covariates, risk factors assessed in the final analyses, and model validation were extracted. In addition, the direction of association of covariates, risk factors in the final model and sub-analyses were also extracted. COVIDENCE Systematic Review Software (Veritas Health Innovation, Melbourne, Australia) was used to facilitate title, abstract, full-text screening and data extraction of the studies [34].

### Risk of bias

Risk of bias in each study was assessed using the Quality In Prognosis Studies (QUIPS) tool checklist [35]. Each study was classified to be of low, moderate or high risk of bias in each of the six domains (study participation, study attrition, prognostic factors, outcome measurements, adjustment for other factors, statistical analysis and reporting). Differences in risk assessments between authors were resolved in discussions between the reviewers.

### Data synthesis

Study characteristics of included papers were summarised. The list of risk factors was presented for discussion with an expert workgroup comprising researchers, palliative care specialists and nephrologists who worked on the grouping of risk factors into different domains. Currently, there are no existing frameworks to guide the categorisation of risk factors of mortality in the ESRD population. Risk factors were grouped based on the domains the risk factors represented, and the groups were aligned with the grouping of risk factors in the primary studies. For example, age, gender, ethnicity, and smoking status describe demographic characteristics or lifestyle choices of an individual and were grouped together in the demographics/lifestyle domain. Readmission and history of hospitalisation are proxy indicators of poor health and were grouped together in the same category. Overall, risk factors were categorised into 13 domains after the discussion.

### Number of papers by risk factors

To provide a comprehensive description of the risk factors that were frequently examined, the number of papers with a reported direction of association of each risk factor with all-cause mortality was tabulated. Papers that did not report the direction of association for the examined risk factor were excluded (Table 1).

**Table 1:** Summary of commonly examined risk factors

Domain	Risk factor	No. of papers	No. of models	No. models which were significant	% models which were significant	Increased mortality risk	Decreased mortality risk
Demographics/ lifestyle	Increasing age	37 [36–71]	64	51	79.7%	100% [36, 40–48, 50–58, 61–66, 68, 70, 71]	-
Demographics/ lifestyle	Female, gender	20 [39, 42–48, 50–53, 56, 57, 61, 62, 66, 67, 72, 73]	32	13	40.6%	100% [42, 44–47, 52, 57, 61, 62]	-
Comorbidities	Presence of diabetes	19 [36, 39, 42–47, 49, 50, 55–57, 66, 67, 69–71, 74]	24	11	45.8%	90.0% [36, 42, 49, 50, 56, 57, 74]	9.1% [46]
Comorbidities	Presence of heart conditions	18 [36, 37, 42–47, 49, 50, 55, 64, 68, 70, 71, 74–76]	27	20	74.1%	95% [36, 37, 43–46, 49, 64, 68, 70, 71]	5% [68]
Biomarkers	Decreasing albumin levels	19 [44–49, 54–56, 61, 64, 66, 67, 71, 73, 77–80]	27	20	74.1%	100% [44–46, 61, 66]	-
Biomarkers	Increasing C-reactive protein	12 [38, 43, 47, 48, 63, 65, 71, 78, 80–83]	27	22	81.5%	100% [38, 43, 47, 48, 65, 78, 82, 83]	-
Biomarkers	Increasing haemoglobin levels	10 [22, 42, 44–47, 55, 56, 67, 80, 84]	29	11	37.9%	-	100% [42, 44–46, 56]
Frailty status	Lower body mass index	11 [42, 44–47, 52, 56, 64, 79]	23	16	69.6%	75% [44–46]	25% [42, 64]
Frailty status	Poor functional status	9 [44–46, 53, 85–89]	20	15	75.0%	100% [44–46, 85–89]	-

### Number of papers by domains

**Fig. 1:**
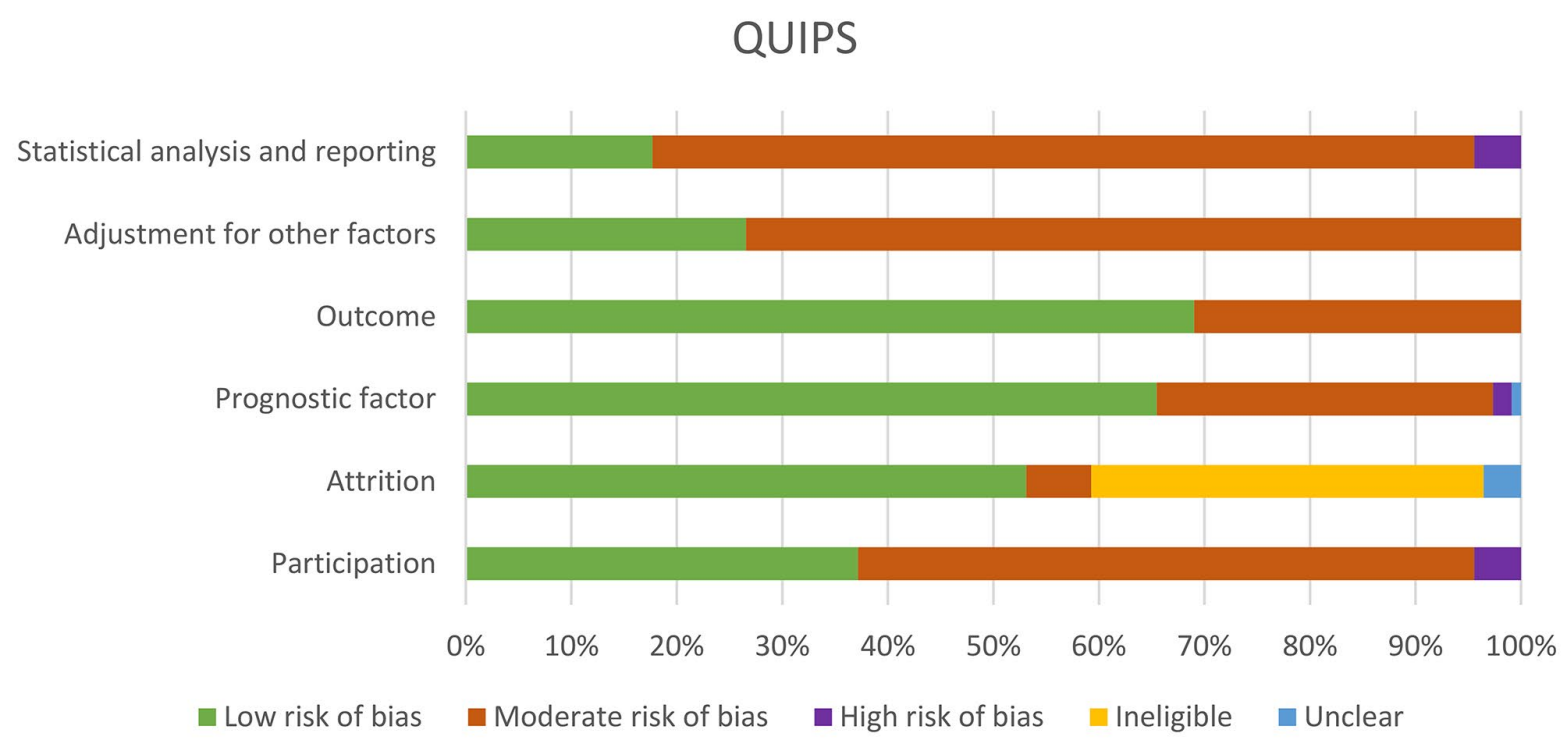
QUIPS

To provide a summary of the domains that were frequently examined, the number of papers with a reported direction of association for each risk factor in the specific domain were tabulated (Fig. 1).

### Percentage of models that reported a significant association between risk factors and mortality

The proportion of models with a reported direction of association where the association was statistically significant was tabulated. The following calculation was performed: the number of models with a reported significant association within a specified domain divided by the total number of models with a reported direction of association within a specified domain multiplied by 100.

## Results

**Fig. 2:**
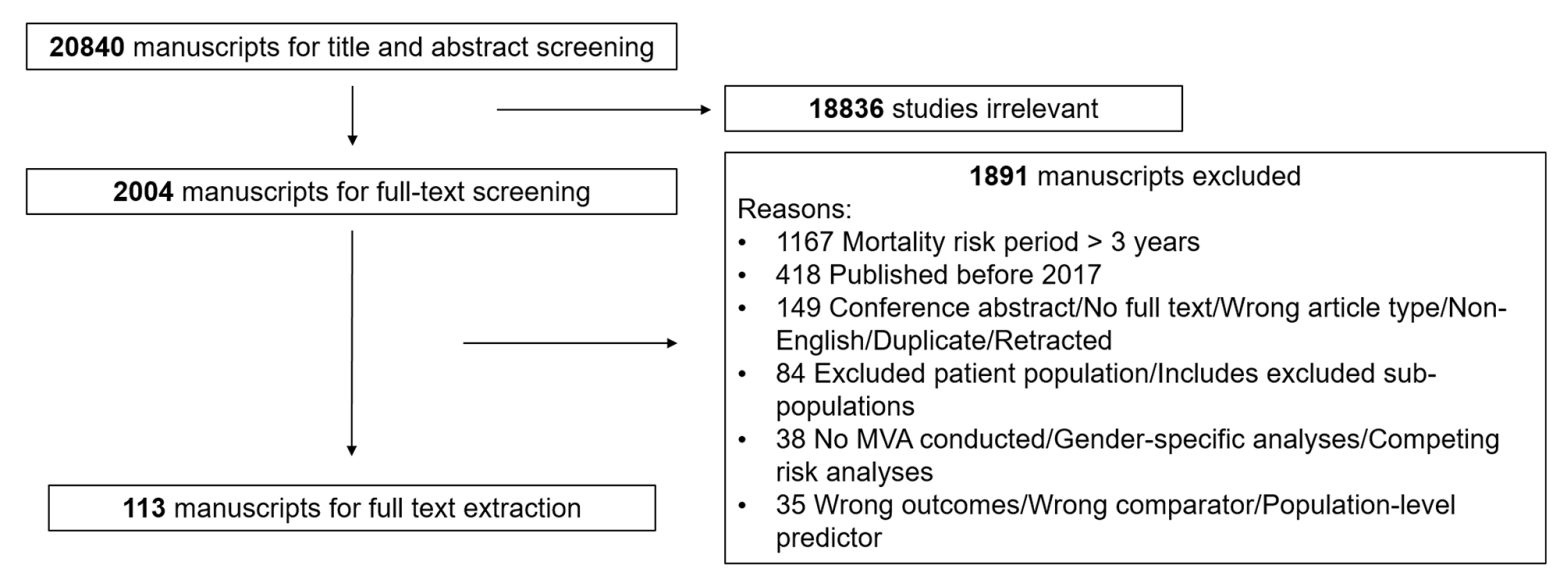
PRISMA flowchart

In this study, 20,840 articles were identified. Of these, 2,004 were included for full text screening. After excluding papers that did not meet the inclusion criteria, 113 papers were included for this review (Fig. 2).

### QUIPS

The majority of papers were rated moderate risk of bias for *statistical analysis and reporting* (88 papers), *adjustment for other factors* (83 papers) and *study participation* (66 papers). The main reason for the assessment of moderate risk was the lack of model diagnostics reporting, limited adjustments on covariates and small sample size (Fig. 1).

### Study characteristics

The study characteristics are described in Table 2. The sample size of the included papers ranged from 30 to 944,650 participants. 94 papers reported the average age of participants (range: 45 years to 87.4 years) and 19 papers reported median age (range: 40 years to 87 years). Of the 113 papers, 6.2% included only peritoneal dialysis (PD) patients, 67.3% included only hemodialysis (HD) patients, with 20.4% of both PD *and* HD patients, whilst the remaining papers featured patients on conservative management or awaiting renal transplant. 11.5% (*n* = 45) of papers reported < 1-year mortality whilst 88.5% (*n* = 347) of papers reported mortality between 1 and 3 years (Table 2). 6 papers conducted validation for the prognostic factors, of which 4 conducted external validation.

**Table 2:** Characteristics of studies

Characteristics	Number of papers (*n* = 113)	Range
Age (years)		
Mean age	94	45-87.4
Median age	19	40–87
Estimated glomerular filtration rate (eGFR) (mL/min/1.73m^2^)		
Mean	15	4.1–14.0
Median	10	4.0-10.1
Not reported	88	NA
Dialysis duration (months)		
Mean	40	12–135
Median	29	15–92
Not reported	44	NA
Mortality rate (%)		
Reported	100	1.3–51
Not reported	13	NA
Sample size of primary papers	113	30–944,650
**Characteristics**	**Number of papers**	**Percentage**
Follow-up time (months)		
Mean reported	21	5-78.1
Median reported	17	9.48–39.8
Maximum reported	71	0.03–240
Not reported	4	NA
Population type		
HD	76	67.3
PD	7	6.2
Mix HD and PD	23	20.4
Diabetic ESRD	1	0.9
Waiting for kidney transplant	1	0.9
General ESRD*	5	4.3
Statistical model		
Cox proportional hazards	90	79.6
Logistic regression	17	15
Modified Poisson regression	3	2.7
Marginal structural Cox model	2	1.8
Lasso penalised logistic regression	1	0.9
Validation		
Internal validation reported	3	2.7
External validation reported	4	3.5
Not performed	106	93.8
**Characteristics**	**Number of models (** ***n*** ** = 392)**	**Percentage**
Mortality risk period		
**< 1 year**	45	11.5
**1–3 years**	347	88.5
**< 1 year**		
1–30 days	12	26.7
> 1 month to ≤ 6 months	26	57.8
> 6 months to < 1 year	7	15.6
**1–3 years**		
1 year	191	55
> 1year − 3 years	156	45

NA: Not applicable; HD: Hemodialysis; PD: Peritoneal diaylysis; *mix of patients on renal replacement therapy or conservative management or patients prior to any treatment/management

### Risk factors

150 risk factors were identified from this review (Supplementary Table 3). Risk factors were categorised into 13 domains (Table 3).

**Table 3:** Overview of domains

Categorisation of domains	Definition
1. Demographics/ lifestyle	Variables that measure demographic characteristics and lifestyle preferences
2. Comorbidities	Variables that reflect co-existing health conditions
3. Intradialytic blood pressure	Variables that measure blood pressure between dialysis session
4. Biomarkers	Variables that provide an objective measure that provides information on the health state on an individual
5. Cardiovascular measurements	Variables that measure cardiovascular function
6. Frailty status	Variables that reflect age-related reduction in physiologic reserve
7. Medications	Variables that describe the type of medications used by patients
8. Treatment related indicators	Variables that describe the treatment patients are undergoing/ have undergone
9. Renal related parameters	Variables that reflect the health status of the kidney
10. Health status	Variables that reflect the overall physical status of patients
11. Cause of ESRD	Variables that indicate the root cause of ESRD
12. Access to healthcare care/ information	Variables that describe an individual’s access to healthcare or information
13. Proxy measures of poor health	Variables that provide an indirect reflection of a patient’s health status

**Fig. 3:**
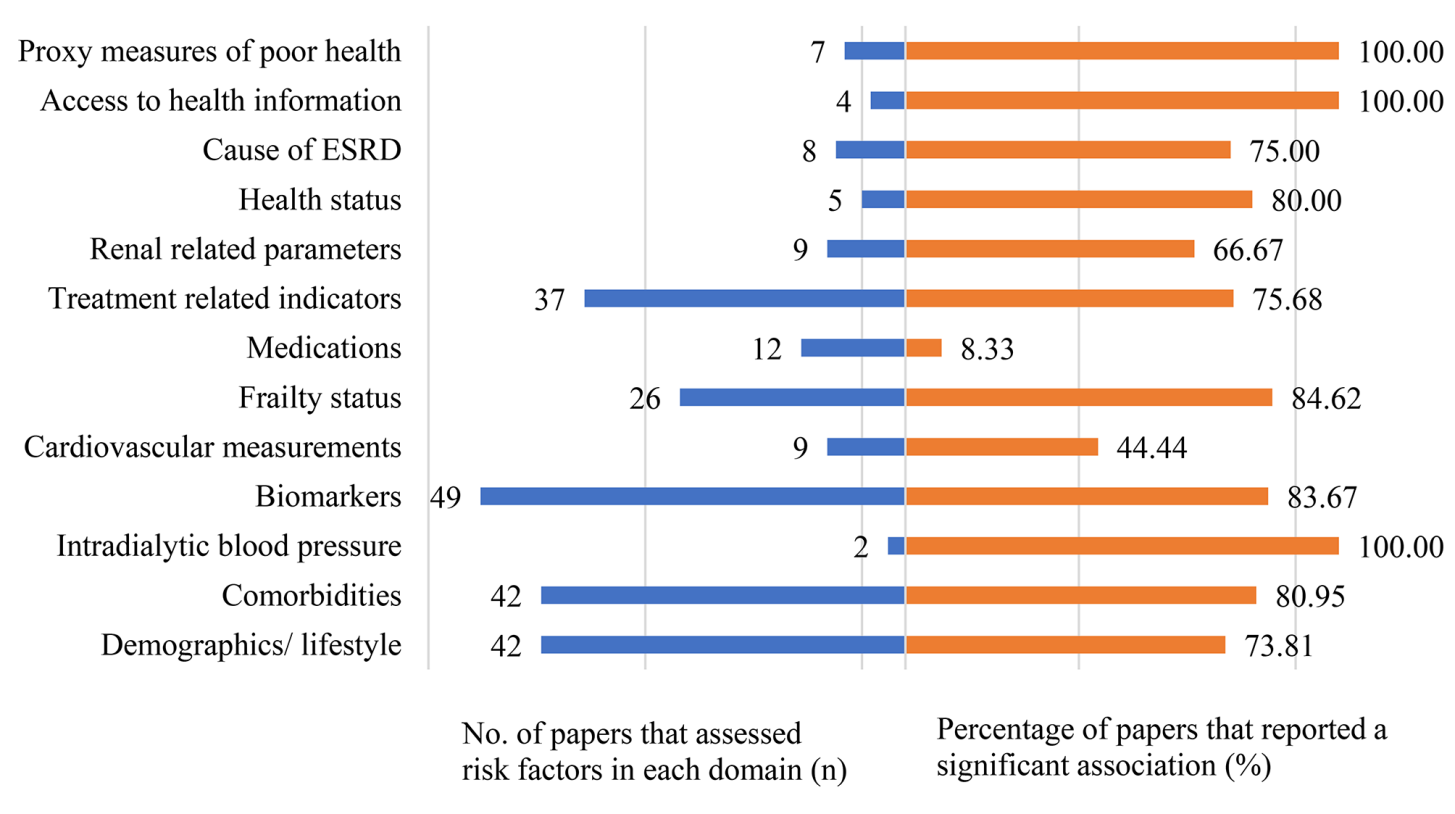
Domains of risk factors assessed for short-term all-cause mortality

Of the 13 domains, biomarkers (49 papers), demographics, lifestyle (42 papers), comorbidities (42 papers) and treatment related indicators (37 papers) were most examined (Fig. 3). Proxy measures of poor health domain, access to health information and intradialytic blood pressure had the highest percentage of papers that reported a significant association with short-term all-cause mortality risk (Fig. 3).

Among the risk factors commonly examined, C-reactive protein (CRP), age, functional status, and albumin levels were more frequently observed to be significantly associated with short-term all-cause mortality (Table 1).

### Demographics and lifestyle

Ninety-nine papers examined the association between demographics and lifestyle with mortality. However, only 42 papers reported the direction of association (Fig. 3). Older age was observed to be significantly associated with a higher mortality risk in 51 models (28 papers). Female gender was observed to have significantly lower risk of mortality compared to the male gender (13 models, 9 papers) (Table 1).

### Comorbidities

Eighty-eight papers explored the association between comorbidities and mortality, of which 42 papers reported the direction of association (Fig. 1). Presence of heart conditions (20 models, 74.1% of the models, 11 papers) and diabetes mellitus (11 models, 45.8% of the models, 7 papers) were significantly associated with a higher mortality risk (Table 1). Possible reasons include, small sample size and similarity in prevalence of diabetes in participants who passed away and those who did not, could explain the lack of statistical significance in association between diabetes status and mortality in some papers.

### Intradialytic blood pressure

Four papers explored the association between intradialytic blood pressure and mortality, with only 2 papers reporting the direction of association (Fig. 3). Among individuals on haemodialysis (*n* = 37,094), it was observed that the presence of intradialytic hypertension increased an individual’s risk of mortality [90]. In another study conducted among 112,013 incident haemodialysis patients, those who experienced episodes of intradialytic hypotension were at increased risk of mortality compared to patients who did not experience any episodes intradialytic hypotension [91]. 

### Biomarkers

Forty-seven papers examined the association between biomarkers and mortality, with direction of association reported in 43 papers (Fig. 3). Albumin (19 papers) and CRP (12 papers) levels were commonly examined in this domain. The papers that investigated the association of albumin with mortality were conducted among patients undergoing dialysis. 12 models (10 papers) analysed albumin as a continuous variable and reported a negative association between albumin and mortality risk. When albumin was analysed as a binary variable (8 models, 5 papers) with serum albumin > = 3.5 mg/dl or 35 g/L as reference, serum albumin levels less than 3.5 mg/dl or 35 g/L were associated with increased risks of mortality. 9 models (6 papers) amongst patients on dialysis consistently reported increased levels of creatinine as being significantly associated with decreased risk of mortality. Papers which did not report a significant association between decreasing albumin and mortality may be due to the small sample sizes or extensive variables included in the adjusted models.

Increased CRP was observed to be significantly associated with increased risk of mortality in 22 models (81.5% of the models, 8 papers) (Table 1).

### Cardiovascular measurements

Fourteen papers explored the association between cardiovascular measurements and mortality, with 9 papers reporting a direction of association with mortality (Fig. 3). Association of cardiac troponin T with mortality was examined in 2 papers, of which, 1 model (1 study) reported significant association with mortality. Specifically, increased serum levels of cardiac troponin T were positively associated with higher mortality risk.

### Frailty status

Fifty-three papers examined the association between frailty and mortality, of which 26 papers reported direction of association (Fig. 3). Functional status (10 papers) and body mass index (BMI) (11 papers) were most frequently examined in this domain. A variety of methods (e.g. impaired mobility, inability to ambulate, inability to transfer, need of assistance with daily activities, independence of daily living, short physical performance battery scores, Barthel index, grip strength) were employed to measure functional status. Despite this, poor functional status was consistently observed to be significantly associated with higher mortality risk (15 models, 100% of the models, 8 papers). 12 models (3 papers) reported that BMI categories < 18.5 kg/m^2^, 18.5-24.99 kg/m^2^, 25-29.9 kg/m^2^ had significantly higher risk of mortality compared to BMI category ≥ 30 kg/m^2^.

### Medications

Twenty-eight papers examined the association between medications and mortality, of which 12 papers reported direction of associations (Fig. 3). Statin use was most frequently examined in this domain (2 papers) and was observed to be associated with a significant decrease in risk of mortality in both papers (36 models).

### Treatment related indicators

Seventy-six papers examined the association between treatment related factors and mortality, of which 37 papers reported direction of association (Fig. 3). In this domain, vascular access type (8 papers) and dialysis vintage (length of time on dialysis) (6 papers) were most frequently examined. For vascular access, across the 17 models (77.2% of the models, 5 papers), it was consistently reported that the presence of central venous catheters were associated with a higher risk of mortality compared to arteriovenous fistulas. On the other hand, only 2 papers (2 models, 100% of models) reported a significant association between increasing dialysis vintage with increased mortality risk.

### Renal related parameters

Twenty papers examined the association between renal related parameters with mortality, of which 9 papers reported direction of association. Blood urea nitrogen (BUN) was and estimated glomerular filtration rate (eGFR) were most frequently examined (3 papers).

### Health status

Twenty papers examined the association between health status and mortality, in which 5 papers reported direction of association (Fig. 3). Health-related QOL and Charlson Comorbidity Index (CCI) were most frequently examined in this domain (2 papers each). Health related QOL, measured either using the 12-item or 36-item Short form survey instrument, was significantly associated with increased mortality risk. Lower levels of physical functioning and emotional health were also associated with increased risk of mortality across 14 models (2 papers). Additionally, lower levels of social functioning were also associated with increased risk of mortality. CCI was only reported to be significantly associated with risk of mortality in 2 models (1 paper), with higher CCI scores being associated with increased risk of mortality.

### Cause of ESRD

Causes of ESRD in association with mortality were examined in 22 papers. Of these, 8 papers reported a direction of association (Fig. 3). Reference groups used varied across the papers included in this review, which resulted in inconsistency in the direction of associations. Diabetes-associated ESRD was most used as the reference group (3 papers). Of these models, hypertension as the primary cause of ESRD had a higher risk of mortality when compared to diabetes as the primary cause of ESRD. On the other hand, glomerulonephritis, cystic or hereditary causes of ESRD had lower risks of mortality when compared to diabetes associated ESRD. When glomerulonephritis-induced ESRD was considered as the reference group, diabetes-, hypertension-, neoplasms/tumours-, pyelonephritis-caused ESRD was associated with increased risk of mortality.

### Access to health care or information

Fifteen papers examined the association between access to health care or information with mortality, of which, 4 papers reported the direction of association (Fig. 3). Access to pre-dialysis nephrology care was most examined (3 papers). Individuals who received pre-dialysis nephrology care had lower risks of mortality (8 models, 3 papers) compared to individuals who did not. This was consistently reported among patients undergoing haemodialysis or peritoneal dialysis.

### Proxy measures of poor health

Twelve papers examined the association between proxy measures of poor health indicators and mortality, of which 7 papers reported the direction of association (Fig. 3). History of hospitalisation was most examined (4 papers) in this domain. Readmission to hospital (2 models, 2 papers) and history of hospitalisation (13 models, 4 papers) were significantly associated with increased mortality risk. The cause of, and time period of hospitalisations varied widely across papers; one paper limited hospitalisations to cardiovascular related or infection related hospitalisations only [46], while Kimmel et al. examined the association of psychiatric diagnoses related hospitalisation [92] and Shah et al. included all causes of hospitalisations [45]. Time points of hospitalisations also varied across papers. Shah et al. examined the association of hospitalisation 2 years prior to onset of ESRD [46] while Ross et al. studied hospitalisations 1 year after the start of dialysis [93]. Despite the variation in causes of hospitalisations and time points of hospitalisations across papers, the association between hospitalisations and mortality was constant. Among patients undergoing dialysis, it was reported that hospitalisations 2 years before onset of ESRD was associated with higher risk of mortality compared to individuals without any hospitalisations [46]. Hospitalisations that occurred between 90 days and 1 year within the start of dialysis were also associated with a higher mortality risk. Similarly, readmissions to hospitals were associated with increased risk of mortality [93]. Compared to individuals with no history of hospitalisations, those with readmissions were at higher risk of mortality after adjusting for covariates [93]. 

## Discussion/ conclusion

This present review examined the risk factors that predicted short-term all-cause mortality for patients with ESRD. Measures of poor health, access to health information and intradialytic blood pressure had the highest percentage of papers reporting a significant association with short-term all-cause mortality risk. Among the risk factors frequently examined, CRP, age, and functional status were observed to be included in a high percentage of models that were significantly associated with short-term all-cause mortality. However, both CRP and functional status are often not routinely collected or documented in the clinic setting. Findings from this review provide useful information to build prognostication frameworks for the ESRD population to aid clinical care decisions with regards to the timely integration of renal supportive care.

The majority of papers included in this review were rated *‘moderate risk of bias’* as they do not report results on model diagnostics and applied limited risk adjustments. Most of the population examined were patients on renal replacement therapy, with a vast majority on haemodialysis. This indicates a research gap amongst patients on peritoneal dialysis or conservative management. Furthermore, whilst papers in this review studied the association between the risk factors with short-term mortality, their prospective prognostic values have not been examined. Further studies will be needed to test and validate their prognostic value during the construction of prognostic risk scores for short-term mortality.

Risk prediction scores covered in this review only included populations undergoing renal replacement therapy. Importantly, these risk scores have not been externally validated nor implemented in clinical settings. Therefore, further research studies are needed to confirm the performance of the risk prediction scores and their clinical utility. Given that palliative care preferences, treatment preferences and receptivity to prognostic risk scores varies according to culture, beliefs and religion, examining the applicability of the risk score in a multi-ethnic Asian population will also be valuable [94]. 

CRP measurements reflect underlying chronic inflammatory processes in dialysis patients, intercurrent clinical events, protein-energy wasting and decreased residual renal function. It is possible that persistent non-specific inflammation, loss of residual renal function and systematic inflammation play a role in increased risk of mortality [95]. Unlike CRP, the precise mechanism to explain the association between physical function and mortality is less clear. It is possible that the lack of physical activity or decreased exercise frequency leads to reduction in muscle mass, with increased risk of systematic inflammation, and mortality in ESRD populations [96, 97]. Importantly, exercise interventions have been shown to improve physical status in individuals with ESRD [98], suggesting the importance of monitoring and maintaining an individual’s engagement in exercise programmes and functional status [99]. 

Despite the established associations of CRP and functional status with risk of mortality, they were not commonly included in the computation of mortality risk scores. CRP was only included in one of the 6 risk scores observed in this review [64, 74, 100–103], and functional status was not included in any of the risk scores. The limited inclusion of these risk factors into predictive risk scores may be explained by the lack of readily available patient information; functional status is not routinely measured in the outpatient clinic or inpatient setting [104] whilst CRP may be measured in the clinic, albeit irregularly [105]. A single time point measurement of CRP may not provide an accurate reflection of the level of inflammation or oxidative stress. Other reasons for the lack of inclusion are the variability in measurement methods and absence of standardised clinical cut-off thresholds for CRP measurements [105–107]. For functional assessment, a wide variability of methods were employed across studies, reflecting the lack of standardisation. Overall, these reasons will influence the clinical applicability of the risk factors to predict risk of mortality. Further research is needed to determine a clinical cut-off for CRP measurements and a standardised measurement for functional status before the adoption of these risk factors into prediction scores for short-term mortality. Other risk factors such as race, history of cancer and CKD aetiology were commonly included in the risk scores observed in this review, but not observed to be associated with short-term all-cause mortality in this review.

It was difficult to ascribe the risk factors highlighted in this review as comparable to the mortality risk factors of patients with CKD stages 3–4. Studies that examined prognostic risk factors for patients with CKD stage 3–4 often predicted mortality for 3 years or more. In these studies, echo-graphic factors, urine protein creatinine ratio, eGFR and malnutrition inflammation scores were reported to be associated with mortality [28]. Considering the different survival trajectories of patients with CKD stage 3–4 and patients with ESRD, different prognostication tools will be required.

There is preliminary evidence to suggest that type of vascular access may be a risk factor of mortality specific to patients undergoing dialysis. However, it was observed that vascular access was only examined in a limited number of papers. Therefore, it was difficult to draw definitive conclusions. More studies will be needed to determine if the inclusion of the risk factor of vascular access will improve the performance of a risk prediction model for short-term mortality.

The strength of this review includes the employment of a robust methodology to conduct and report the findings of results based on established scoping review framework, and provision of a comprehensive summary of risk factors for ESRD patients for short-term all-cause mortality. Inclusion of a quality appraisal using a well published tool QUIPS, helped to interpret the robustness of the study methodology and the results. However, this review is not without limitations. Given that this study was a scoping review, it was not possible to standardise the definitions of various risk factors. Nonetheless, despite inconsistent definitions for certain risk factors (e.g., hospitalisations, functional status), consistent associations with mortality were observed. This review only included primary papers with mortality risk periods of 3 years and less and may have inadvertently excluded papers without a clear description of mortality risk period or follow up period. However, the aim of this review was to identify and summarise risk factors for short-term all-cause mortality to facilitate conversations regarding treatment options, goals of care and advance care planning. Hence, the inclusion criteria for this scoping review is unlikely to result in a selection bias. Majority of the papers in this review reported mortality within 1–3 years (mortality within 1–3 years: *n* = 347 models, 88.5%; mortality < 1 year: *n* = 45 models, 11.5%). We performed a preliminary sub-analysis by splitting the outcomes to (1) mortality < 1 year and (2) mortality within 1–3 years. In this preliminary sub-analysis, we observed that biomarkers (7 papers), comorbidities (6 papers) and treatment related indicators (4 papers) were most assessed for mortality < 1 year (Supplementary Fig. 1). On the other hand, biomarkers (43 papers), demographics/lifestyle (40 papers) and comorbidities (38 papers) were most assessed for mortality at 1–3 years (Supplementary Fig. 2). Further studies will be needed to compare the risk factors across the mortality period within 1–3 years and mortality at < 1 year.

In this review, we were unable to compare differences in risk factors between patients undergoing haemodialysis and peritoneal dialysis due to the small number of papers that focused solely on peritoneal dialysis patients (*n* = 7). Papers that were included in this review did not account for dialysis related variables such as weekly treatment time or compared mortality risk between haemodialysis and hemodiafiltration. As such, we were unable to describe the associations between these variables with mortality.

In this review, it was observed that there is a broad spectrum of factors are associated with 3-year mortality in ESRD patients. Further studies are needed to examine the incremental predictive value of risk factors of mortality. In addition, to ensure the clinical utility of future predictive models, clear clinical cut-offs with routine, standardised measurements of the required variables will be needed. Following the development of the prognostic models, examining the implementation process (e.g., acceptability to patients, caregivers and clinicians) will also be crucial to understand the impact of the models on treatment preferences and receptivity. Finally, understanding the difference in risk factors between patients undergoing haemodialysis and peritoneal dialysis will further knowledge of the need of different prognostic models for the two treatment populations.

In conclusion, this study has found multiple factors which influence short-term all-cause mortality in patients with ESRD. While there are consistent findings for CRP and functional status, evidence for type of vascular access is insufficient. Commonly examined risk factors observed from this review may be used to build a general prognostic model for patients with ESRD, with the addition of treatment- specific risk factors to enhance accuracy. Used together with needs assessment tools (e.g., palliative care indicators tool), renal specific prognostic models can be used to identify people with deteriorating health for assessment of unmet needs for supportive and palliative care. Critically, this will help clinicians systematically identify patients at risk of deterioration, ensuring timely assessment of needs and facilitation of treatment preferences and goals of care.

### Electronic supplementary material

Below is the link to the electronic supplementary material.

Supplementary Material 1

## Data Availability

All data generated and/or analysed during the current study are included in the published article and it supplementary information files.
