# Supplementary material for: Risk factors for short-term all-cause mortality in patients with end stage renal disease: a scoping review
Source: BMC Nephrol. 2024 Feb 27;25:71. doi: 10.1186/s12882-024-03503-3 (PMC10900550; doi:10.1186/s12882-024-03503-3)
Supplement: Supplementary file 1 — Supplementary Material 1 [file 12882_2024_3503_MOESM1_ESM.docx]

**Risk Factors for Short-Term All-Cause Mortality in Patients with End Stage Renal Disease: A Scoping Review**

Wanfen Yip^1^, Sheryl Hui Xian Ng ^1^, Palvinder Kaur^1^, Pradeep Paul George^1^, Jennifer Huey Chen Guan ^2^, Guozhang Lee ^3^, Timothy Jee Kam Koh ^4^, Woan Shin Tan^1,5^ *, Allyn Yin Mei Hum ^2, 6^*

*Joint senior authors

1. Health Services & Outcomes Research, National Healthcare Group, Singapore

2. Department of Palliative Medicine, Tan Tock Seng Hospital, Singapore

3. Department of Internal Medicine, Singapore General Hospital, Singapore

4. Department of Renal Medicine, Tan Tock Seng Hospital, Singapore.

5. Geriatric Education and Research Institute, Singapore

6. The Palliative Care Centre for Excellence in Research and Education, Dover Park Hospice, Singapore

Supplementary table 1: Study eligibility criteria

| **Study Characteristics** | **Inclusion Criteria** | **Exclusion Criteria** |
| --- | --- | --- |
| Type of publication | - Peer-reviewed journal articles, PhD Thesis, grey literature | - Editorials, letters, commentaries, abstracts with insufficient information, reviews, genetic studies based on or including animals |
| Study population | - Populations with end-stage renal disease (eGFR <15/ renal replacement therapy - Populations with end-stage renal disease and diabetes - Populations with end-stage renal disease and ischaemic heart disease/ heart failure - Population of interest who is undergoing dialysis with infection - Population of interest and undergone vascular access procedures | - Non adult population (< 18 years old) - Populations with concurrent cancer, auto-immune disease, acute (e.g., COVID)/chronic conditions (e.g., stroke), kidney transplant - All-cause mortality > 3 years |
| Study design | - Quantitative studies which employed prospective, retrospective, longitudinal, RCT, and cross-sectional designs - Studies that performed external validation and reported variables or referenced the primary study | - Qualitative studies - Cost-effectiveness studies |
| Intervention | - Treatment or intervention studies | - N.A. |
| Analysis | - Studies that conducted multivariable analysis - Studies that analyzed any factors associated with or predicted mortality/survival - Studies reporting subgroup-specific multivariate analysis for populations | - Studies that conducted univariable analysis only - Studies that conducted competing risk analyses |
| Outcome | - All-cause mortality within 3 years   - For studies with no defined mortality risk period, maximum follow-up time must fall within 3 years   - If studies did not report maximum follow-up time, mean + 3 standard deviations, or median + 2 interquartile range must fall within 3 years | - Composite outcomes (e.g. death and hospitalisation) - Only non-mortality outcomes assessed |
| Language | - English | - Other languages |
| Geography | - Worldwide | - N.A. |

Supplementary table 2: Search strategy

| PubMed | 1. ("kidney failure, chronic"[Mesh:NoExp]) OR ("renal insufficiency"[Mesh:NoExp]) 2. "chronic kidney failure"[Title/Abstract] OR ESRD[Title/Abstract] OR "decreased renal function"[Title/Abstract] OR "end-stage kidney disease"[Title/Abstract] OR "end stage kidney disease"[Title/Abstract] OR "end-stage renal disease"[Title/Abstract] OR "end stage renal disease"[Title/Abstract] OR "renal failure"[Title/Abstract] OR "chronic renal disease"[Title/Abstract] OR ESKD[Title/Abstract] OR "chronic kidney disease stage 5"[Title/Abstract] OR "stage 5 chronic kidney disease"[Title/Abstract] OR CKD5[Title/Abstract] 3. #1 OR #2 4. renal dialysis[MeSH Terms] 5. dialysis[Title/Abstract] OR haemodialysis[Title/Abstract] OR hemodialysis[Title/Abstract] OR "renal replacement therapy"[Title/Abstract] OR haemodiafiltration[Title/Abstract] OR hemodiafiltration[Title/Abstract] OR "conservative management"[Title/Abstract] OR "conservative treatment"[Title/Abstract] OR "nondialytic therapy"[Title/Abstract] OR "non-dialytic therapy"[Title/Abstract] OR "non dialytic therapy"[Title/Abstract] OR "renal supportive care"[Title/Abstract] OR "incident dialysis"[Title/Abstract] OR "maintenance dialysis"[Title/Abstract] OR "maintenance hemodialysis"[Title/Abstract] OR "peritoneal dialysis"[Title/Abstract] OR "continuous ambulatory peritoneal dialysis"[Title/Abstract] 6. #4 OR #5 7. #3 AND #6 8. "survival analysis"[Mesh:NoExp] OR "Kaplan-Meier Estimate"[Mesh:NoExp] OR "proportional hazards models"[Mesh:NoExp] OR "survival rate"[Mesh:NoExp] OR "life expectancy"[Mesh:NoExp] OR "mortality"[Mesh:NoExp] OR "fatal outcome"[Mesh:NoExp] OR "hospital mortality"[Mesh:NoExp] OR "prognosis"[Mesh:NoExp] OR "medical futility"[MeSH Terms] OR "nomograms"[MeSH Terms] OR "logistic models"[Mesh:NoExp] 9. "death rate"[Title/Abstract] OR "death rates"[Title/Abstract] OR "mortality"[Title/Abstract] OR "survival rate"[Title/Abstract] OR "survival rates"[Title/Abstract] OR "death risk"[Title/Abstract] OR "death risks"[Title/Abstract] OR "life expectancy"[Title/Abstract] OR "survival analysis"[Title/Abstract] OR "survival"[Title/Abstract] OR "prognosis"[Title/Abstract] OR "prognostic factor"[Title/Abstract] OR "prognostic factors"[Title/Abstract] OR "prediction"[Title/Abstract] OR "predictions"[Title/Abstract] OR "prognostic model"[Title/Abstract] OR "prognostic models"[Title/Abstract] OR "prediction model"[Title/Abstract] OR "prediction models"[Title/Abstract] OR "survival model"[Title/Abstract] OR "survival models"[Title/Abstract] OR "death"[Title/Abstract] OR "dying"[Title/Abstract] OR "logistic model"[Title/Abstract] OR "logistic models"[Title/Abstract] OR "logistic regression"[Title/Abstract] OR "logit model"[Title/Abstract] OR "logit models"[Title/Abstract] 10. #8 OR #9 11. "epidemiologic studies"[MeSH Terms:noexp] OR "case control studies"[MeSH Terms] OR "cohort studies"[MeSH Terms] OR "case control"[Text Word] OR "cohort study"[Text Word] OR "cohort studies"[Text Word] OR "cohort analy*"[Text Word] OR "Follow up study"[Text Word] OR "Follow up studies"[Text Word] OR "observational study"[Text Word] OR "observational studies"[Text Word] OR "Longitudinal"[Text Word] OR "Retrospective"[Text Word] OR "Cross sectional"[Text Word] OR "cross sectional studies"[MeSH Terms:noexp] 12. "randomized controlled trials as topic"[MeSH Terms:noexp] OR "Randomized controlled trial"[MeSH Terms:noexp] OR "random allocation"[MeSH Terms:noexp] OR "double b 13. d method"[MeSH Terms:noexp] OR "single blind method"[MeSH Terms:noexp] OR "Clinical trial"[MeSH Terms:noexp] OR clinical trials as topic[MeSH Terms] 14. ("Clinic* trial*1"[Text Word] OR "singl* blind*3"[Text Word] OR "singl* mark*3"[Text Word] OR "doubl* blind*3"[Text Word] OR "doubl* mark*3"[Text Word] OR "treb* blind*3"[Text Word] OR "treb* mark*3"[Text Word] OR "tripl* blind*3"[Text Word] OR "tripl* mask*3"[Text Word] OR placebo*[Text Word] OR "randomly allocated"[Text Word] OR "allocated at random"[Text Word]) OR ("placebos"[MeSH Terms:noexp]) 15. #11 OR #12 OR #13 16. (((("case report"[Text Word]) OR (letter[MeSH Terms:noexp])) OR (Historical article[MeSH Terms:noexp])) OR (Review of reported cases[Publication Type])) OR (Review, multicase[Publication Type]) 17. #14 NOT #15 18. #3 AND #6 AND #10 AND #16 |
| --- | --- |
| EMBASE | Database: Embase <1974 to 2020 November 09>  Search Strategy:  --------------------------------------------------------------------------------   1. chronic kidney failure/ or kidney failure/ (232065) 2. ("chronic kidney failure" or ESRD or "decreased renal function" or "End-Stage Kidney Disease" or "End Stage Kidney Disease" or ("End-Stage" adj2 "Renal Disease") or ("End Stage" adj2 "Renal Disease") or "Renal Failure" or "Chronic Renal Disease" or ESKD or "Chronic Kidney Disease Stage 5" or "Stage 5 Chronic Kidney Disease" or CKD5).ab,ti,tw. (186256) 3. 1 or 2 (320309) 4. exp hemodialysis/ (112472) 5. (dialysis or h?emodialysis or "renal replacement therapy" or h?emodiafiltration or (conservative adj2 management) or "conservative treatment" or "nondialytic therapy" or "non-dialytic therapy" or "non dialytic therapy" or "renal supportive care" or "incident dialysis" or "maintenance dialysis" or "maintenance hemodialysis" or "peritoneal dialysis" or "continuous ambulatory peritoneal dialysis").ab,ti,tw. (286937) 6. 4 or 5 (312057) 7. 3 and 6 (98136) 8. survival analysis/ or kaplan meier method/ or proportional hazards model/ or survival rate/ or life expectancy/ or mortality/ or fatality/ or hospital mortality/ or prognosis/ or exp treatment outcome/ or nomogram/ or statistical model/ (3302434) 9. ("death rate?" or mortality or "survival rate?" or "death risk?" or "life expectancy" or "survival analysis" or survival or prognosis or "prognostic factor?" or prediction? or "prognostic model?" or "prediction model?" or "survival model?" or death or dying or "logistic model?" or "logistic regression" or "logit model?").ab,ti,tw. (4081965) 10. 8 or 9 (5840606) 11. epidemiology/ or exp case control study/ or cohort analysis/ or cross-sectional study/ or (Case control or (cohort adj (study or studies)) or Cohort analy$ or (Follow up adj (study or studies)) or (observational adj (study or studies)) or Longitudinal or Retrospective or Cross sectional).tw. (2769567) 12. "randomized controlled trial (topic)"/ or randomized controlled trial/ or randomization/ or double blind procedure/ or single blind procedure/ or clinical trial/ or exp "clinical trial (topic)"/ (1738336) 13. ((clinic$ adj trial$1) or ((singl$ or doubl$ or treb$ or tripl$) adj (blind$3 or mask$3))).tw. or placebo/ or Placebo$.tw. or Randomly allocated.tw. or (allocated adj2 random).tw. (1036237) 14. 11 or 12 or 13 (4722455) 15. Case report.tw. or letter/ or Review of reported cases.pt. or Review, multicase.pt. (1511187) 16. 14 not 15 (4643392) 17. 3 and 6 and 10 and 16 (15038) 18. limit 17 to (human and english language and yr="2000 -Current" and (adult <18 to 64 years> or aged <65+ years>)) (8914) |
| Medline | Database: Ovid MEDLINE(R) and Epub Ahead of Print, In-Process & Other Non-Indexed Citations, Daily and Versions(R) <1946 to November 09, 2020>  Search Strategy:  --------------------------------------------------------------------------------   1. Kidney Failure, Chronic/ or Renal Insufficiency/ (109943) 2. ("chronic kidney failure" or ESRD or "decreased renal function" or "End-Stage Kidney Disease" or "End Stage Kidney Disease" or ("End-Stage" adj2 "Renal Disease") or ("End Stage" adj2 "Renal Disease") or "Renal Failure" or "Chronic Renal Disease" or ESKD or "Chronic Kidney Disease Stage 5" or "Stage 5 Chronic Kidney Disease" or CKD5).ab,ti,tw. (128177) 3. 1 or 2 (187086) 4. exp Renal Dialysis/ (114631) 5. (dialysis or h?emodialysis or "renal replacement therapy" or h?emodiafiltration or (conservative adj2 management) or "conservative treatment" or "nondialytic therapy" or "non-dialytic therapy" or "non dialytic therapy" or "renal supportive care" or "incident dialysis" or "maintenance dialysis" or "maintenance hemodialysis" or "peritoneal dialysis" or "continuous ambulatory peritoneal dialysis").ab,ti,tw. (206493) 6. 4 or 5 (229308) 7. 3 and 6 (76630) 8. survival analysis/ or kaplan-meier estimate/ or proportional hazards models/ or Survival Rate/ or Life Expectancy/ or mortality/ or fatal outcome/ or hospital mortality/ or prognosis/ or exp medical futility/ or exp nomograms/ or Logistic Models/ (1070176) 9. ("death rate?" or mortality or "survival rate?" or "death risk?" or "life expectancy" or "survival analysis" or survival or prognosis or "prognostic factor?" or prediction? or "prognostic model?" or "prediction model?" or "survival model?" or death or dying or "logistic model?" or "logistic regression" or "logit model?").ab,ti,tw. (2891980) 10. 8 or 9 (3297966) 11. Epidemiologic studies/ or exp case control studies/ or exp cohort studies/ or Case control.tw. or (cohort adj (study or studies)).tw. or Cohort analy$.tw. or (Follow up adj (study or studies)).tw. or (observational adj (study or studies)).tw. or Longitudinal.tw. or Retrospective.tw. or Cross sectional.tw. or Cross-sectional studies/ (3094290) 12. Randomized controlled trials as Topic/ or Randomized controlled trial/ or Random allocation/ or Double blind method/ or Single blind method/ or Clinical trial/ or exp Clinical Trials as Topic/ (1190067) 13. ((clinic$ adj trial$1) or ((singl$ or doubl$ or treb$ or tripl$) adj (blind$3 or mask$3))).tw. or Placebos/ or Placebo$.tw. or Randomly allocated.tw. or (allocated adj2 random).tw. (650227) 14. 11 or 12 or 13 (4257228) 15. Case report.tw. or Letter/ or Historical article/ or Review of reported cases.pt. or Review, multicase.pt. (1771825) 16. 14 not 15 (4155113) 17. 3 and 6 and 10 and 16 (12810) 18. limit 17 to (english language and humans and yr="2000 -Current" and "all adult (19 plus years)") (8623) |
| CINAHL | 1. (MH "Renal Insufficiency") OR (MH "Kidney Failure, Chronic") 2. TI ( "chronic kidney failure" or ESRD or "decreased renal function" or "End-Stage Kidney Disease" or "End Stage Kidney Disease" or ("End- Stage" N2 "Renal Disease") or ("End Stage" N2 "Renal Disease") or "Renal Failure" or "Chronic Renal Disease" or ESKD or "Chronic Kidney Disease Stage 5" or "Stage 5 Chronic Kidney Disease" or CKD5 ) OR AB ( "chronic kidney failure" or ESRD or "decreased renal function" or "End-Stage Kidney Disease" or "End Stage Kidney Disease" or ("End- Stage" N2 "Renal Disease") or ("End Stage" N2 "Renal Disease") or "Renal Failure" or "Chronic Renal Disease" or ESKD or "Chronic Kidney Disease Stage 5" or "Stage 5 Chronic Kidney Disease" or CKD5 ) 3. S1 OR S2 4. TI ( dialysis or h#emodialysis or "renal replacement therapy" or h#emodiafiltration or (conservative N2 management) or "conservative treatment" or "nondialytic therapy" or "non-dialytic therapy" or "non dialytic therapy" or "renal supportive care" or "incident dialysis" or "maintenance dialysis" or "maintenance hemodialysis" or "peritoneal dialysis" or "continuous ambulatory peritoneal dialysis" ) OR AB ( dialysis or h#emodialysis or "renal replacement therapy" or h#emodiafiltration or (conservative N2 management) or "conservative treatment" or "nondialytic therapy" or "non-dialytic therapy" or "non dialytic therapy" or "renal supportive care" or "incident dialysis" or "maintenance dialysis" or "maintenance hemodialysis" or "peritoneal dialysis" or "continuous ambulatory peritoneal dialysis" ) 5. #3 AND #4 6. (MH "Survival Analysis") OR (MH "Kaplan-Meier Estimator") OR (MH "Cox Proportional Hazards Model") OR (MH "Life Expectancy") OR (MH "Hospital Mortality") OR (MH "Mortality") OR (MH "Fatal Outcome") OR (MH "Prognosis") OR (MH "Medical Futility") 7. TI ( "death rate#" or mortality or "survival rate#" or "death risk#" or "life expectancy" or "survival analysis" or survival or prognosis or "prognostic factor#" or prediction# or "prognostic model#" or "prediction model#" or "survival model#" or death or dying or "logistic model#" or "logistic regression" or "logit model#" ) OR AB ( "death rate#" or mortality or "survival rate#" or "death risk#" or "life expectancy" or "survival analysis" or survival or prognosis or "prognostic factor#" or prediction# or "prognostic model#" or "prediction model#" or "survival model#" or death or dying or "logistic model#" or "logistic regression" or "logit model#" ) 8. #6 OR #7 9. (MH "Epidemiological Research") OR (MH "Case Control Studies+") OR (MH "Prospective Studies+") OR TI "Case control" OR AB "Case control" OR TI ( cohort N (study or studies) ) OR AB ( cohort N (study or studies) ) OR TI "Cohort analy*" OR AB "Cohort analy*" OR TI ( ("Follow up" N (study or studies)) or (observational N (study or studies)) or Longitudinal or Retrospective or "Cross sectional" ) OR AB ( ("Follow up" N (study or studies)) or (observational N (study or studies)) or Longitudinal or Retrospective or "Cross sectional" ) OR (MH "Cross Sectional Studies") 10. (MH "Randomized Controlled Trials") OR (MH "Random Assignment") OR (MH "Double-Blind Studies") OR (MH "Single-Blind Studies") OR (MH "Clinical Trials") 11. TI ( ((clinic* N trial*1) or ((singl* or doubl* or treb* or tripl*) N (blind*3 or mask*3))) or Placebo* or "Randomly allocated" or (allocated N2 random) ) OR AB ( ((clinic* N trial*1) or ((singl* or doubl* or treb* or tripl*) N (blind*3 or mask*3))) or Placebo* or "Randomly allocated" or (allocated N2 random) ) OR (MH "Placebos") 12. #9 OR #10 OR #11 13. TI "Case report" OR AB"Case report" OR PT"Review of reportedcases" OR PT "Review,multicase" 14. #12 NOT#13 15. #5 AND#8 AND#14 |
| Cochrane | 1. MeSH descriptor: [Kidney Failure, Chronic] this term only 2. MeSH descriptor: [Renal Insufficiency] this term only 3. ("chronic kidney failure" or ESRD or "decreased renal function" or "End-Stage Kidney Disease" or "End Stage Kidney Disease" or ("End-Stage" NEAR/2 "Renal Disease") or ("End Stage" NEAR/2 "Renal Disease") or "Renal Failure" or "Chronic Renal Disease" or ESKD or "Chronic Kidney Disease Stage 5" or "Stage 5 Chronic Kidney Disease" or CKD5):ti,ab,kw (Word variations have been searched) 4. #1 OR #2 OR #3 5. MeSH descriptor: [Renal Dialysis] explode all trees 6. (dialysis or h?emodialysis or "renal replacement therapy" or h?emodiafiltration or (conservative NEAR/2 management) or "conservative treatment" or "nondialytic therapy" or "non-dialytic therapy" or "non dialytic therapy" or "renal supportive care" or "incident dialysis" or "maintenance dialysis" or "maintenance hemodialysis" or "peritoneal dialysis" or "continuous ambulatory peritoneal dialysis"):ti,ab,kw (Word variations have been searched) 7. #5 OR #6 8. #4 AND #7 9. MeSH descriptor: [Survival Analysis] this term only 10. MeSH descriptor: [Kaplan-Meier Estimate] this term only 11. MeSH descriptor: [Proportional Hazards Models] this term only 12. MeSH descriptor: [Survival Rate] this term only 13. MeSH descriptor: [Life Expectancy] this term only 14. MeSH descriptor: [Mortality] this term only 15. MeSH descriptor: [Fatal Outcome] this term only 16. MeSH descriptor: [Hospital Mortality] this term only 17. MeSH descriptor: [Prognosis] this term only 18. MeSH descriptor: [Nomograms] explode all trees 19. MeSH descriptor: [Logistic Models] this term only 20. MeSH descriptor: [Medical Futility] explode all trees 21. ("death rate?" or mortality or "survival rate?" or "death risk?" or "life expectancy" or "survival analysis" or survival or prognosis or "prognostic factor?" or prediction? or "prognostic model?" or "prediction model?" or "survival model?" or death or dying or "logistic model?" or "logistic regression" or "logit model?"):ti,ab,kw (Word variations have been searched) 22. #9 OR #10 OR #11 OR #12 OR #13 OR #14 OR #15 OR #16 OR #17 OR #18 OR #19 OR #20 OR #21 23. MeSH descriptor: [Epidemiologic Studies] this term only 24. MeSH descriptor: [Case-Control Studies] explode all trees 25. MeSH descriptor: [Cohort Studies] explode all trees 26. MeSH descriptor: [Cross-Sectional Studies] this term only 27. ("Case control" or "cohort study" OR "cohort studies" or "Cohort analy*" or "Follow up study" OR "follow up studies" or "observational study" OR "observational studies" or Longitudinal or Retrospective or "Cross sectional"):ti,ab,kw (Word variations have been searched) 28. MeSH descriptor: [Randomized Controlled Trials as Topic] this term only 29. MeSH descriptor: [Randomized Controlled Trial] this term only 30. MeSH descriptor: [Random Allocation] this term only 31. MeSH descriptor: [Double-Blind Method] this term only 32. MeSH descriptor: [Single-Blind Method] this term only 33. MeSH descriptor: [Clinical Trial] this term only 34. MeSH descriptor: [Clinical Trials as Topic] explode all trees 35. MeSH descriptor: [Placebos] this term only 36. (((clinic* NEAR trial*1) or ((singl* or doubl* or treb* or tripl*) NEAR (blind*3 or mask*3))) or Placebo* or "Randomly allocated" or (allocated NEAR/2 random)):ti,ab,kw 37. #23 OR #24 OR #25 OR #26 OR #27 OR #28 OR #29 OR #30 OR #31 OR #32 OR #33 OR #34 OR #35 OR 592187 38. (Case report):ti,ab,kw 39. MeSH descriptor: [Letter] this term only 40. MeSH descriptor: [Historical Article] this term only 41. (Review of reported cases):pt 42. (Review, multicase):pt 43. #38 OR #39 OR #40 OR #41 OR #42 44. #37 NOT #43 45. #8 AND #22 AND #44 with Cochrane Library publication date Between Jan 2000 and Dec 2020, in Cochrane Reviews, Cochrane Protocols, Clinical Answers, Editorials, Special Collections (Word variations have been searched) 46. #8 AND #22 AND #44 with Publication Year from 2000 to 2020, in Trials (Word variations have been searched) |
| Web of Science | 1. TS=("chronic kidney failure" or ESRD or "decreased renal function" or "End-Stage Kidney Disease" or "End Stage Kidney Disease" or 2. ("End-Stage" NEAR/2 "Renal Disease") or ("End Stage" NEAR/2 "Renal Disease") or "Renal Failure" or "Chronic Renal Disease" or ESKD or "Chronic 3. Kidney Disease Stage 5" or "Stage 5 Chronic Kidney Disease" or CKD5 OR "renal insufficiency") 4. Indexes=SCI-EXPANDED, SSCI, A&HCI, CPCI-S, CPCI-SSH, BKCI-S, BKCI-SSH, ESCI Timespan=All years 5. TS=(dialysis or h$emodialysis or "renal replacement therapy" or h$emodiafiltration or (conservative NEAR/2 management) or "conservative treatment" 6. or "nondialytic therapy" or "non-dialytic therapy" or "non dialytic therapy" or "renal supportive care" or "incident dialysis" or "maintenance dialysis" 7. or "maintenance hemodialysis" or "peritoneal dialysis" or "continuous ambulatory peritoneal dialysis") 8. Indexes=SCI-EXPANDED, SSCI, A&HCI, CPCI-S, CPCI-SSH, BKCI-S, BKCI-SSH, ESCI Timespan=All years 9. #1 AND #2 10. Indexes=SCI-EXPANDED, SSCI, A&HCI, CPCI-S, CPCI-SSH, BKCI-S, BKCI-SSH, ESCI Timespan=All years 11. TS=("death rate$" or mortality or "survival rate$" or "death risk$" or "life expectancy" or "survival analysis" or survival or prognosis or "prognostic 12. factor$" or prediction$ or "prognostic model$" or "prediction model$" or "survival model$" or death or dying or "logistic model$" or "logistic 13. regression" or "logit model$") 14. Indexes=SCI-EXPANDED, SSCI, A&HCI, CPCI-S, CPCI-SSH, BKCI-S, BKCI-SSH, ESCI Timespan=All years 15. TS=("Case control" or (cohort NEAR (study or studies) ) or Cohort analy* or ("Follow up" NEAR (study or studies) ) or 16. (observational NEAR (study or studies) ) or Longitudinal or Retrospective or "Cross sectional") 17. Indexes=SCI-EXPANDED, SSCI, A&HCI, CPCI-S, CPCI-SSH, BKCI-S, BKCI-SSH, ESCI Timespan=All years 18. TS=(((clinic* NEAR trial*1) or ((singl* or doubl* or treb* or tripl*) NEAR (blind*3 or mask*3) )) or Placebo* or "Randomly allocated" or 19. (allocated NEAR/2 random) ) 20. Indexes=SCI-EXPANDED, SSCI, A&HCI, CPCI-S, CPCI-SSH, BKCI-S, BKCI-SSH, ESCI Timespan=All years 21. #5 OR #6 22. Indexes=SCI-EXPANDED, SSCI, A&HCI, CPCI-S, CPCI-SSH, BKCI-S, BKCI-SSH, ESCI Timespan=All years 23. TS=("Case report") 24. Indexes=SCI-EXPANDED, SSCI, A&HCI, CPCI-S, CPCI-SSH, BKCI-S, BKCI-SSH, ESCI Timespan=All years 25. #7 NOT #8 26. Indexes=SCI-EXPANDED, SSCI, A&HCI, CPCI-S, CPCI-SSH, BKCI-S, BKCI-SSH, ESCI Timespan=All years 27. #3 AND #4 AND #9 28. Indexes=SCI-EXPANDED, SSCI, A&HCI, CPCI-S, CPCI-SSH, BKCI-S, BKCI-SSH, ESCI Timespan=All years 29. #3 AND #4 AND #9 30. Refined by: PUBLICATION YEARS: ( 2020 OR 2012 OR 2004 OR 2019 OR 2011 OR 2003 OR 2018 OR 2010 OR 2002 OR 2017 OR 2009 OR 2001 OR 2016 OR 2008 OR 2000 31. OR 2015 OR 2007 OR 2014 OR 2006 OR 2013 OR 2005 ) 32. Indexes=SCI-EXPANDED, SSCI, A&HCI, CPCI-S, CPCI-SSH, BKCI-S, BKCI-SSH, ESCI Timespan=All years 33. #3 AND #4 AND #9 34. Refined by: PUBLICATION YEARS: ( 2020 OR 2012 OR 2004 OR 2019 OR 2011 OR 2003 OR 2018 OR 2010 OR 2002 OR 2017 OR 2009 OR 2001 OR 2016 OR 2008 OR 2000 35. OR 2015 OR 2007 OR 2014 OR 2006 OR 2013 OR 2005 ) AND LANGUAGES: ( ENGLISH ) 36. Indexes=SCI-EXPANDED, SSCI, A&HCI, CPCI-S, CPCI-SSH, BKCI-S, BKCI-SSH, ESCI Timespan=All years |

Supplementary table 3: Risk factors associated with short-term mortality all-cause mortality

| **Domain** | **Prognostic factor** | **No. of papers** | **No. of models*** | **No. models were significant** | **% of models were significant** | **Increased mortality risk** | **Decreased mortality risk** |
| --- | --- | --- | --- | --- | --- | --- | --- |
| Demographics/ lifestyle | Ethnicity | 8[[1-8](#_ENREF_1)] | 37 | 35 | 94.6% | 14.3%[[3](#_ENREF_3), [5](#_ENREF_5), [8](#_ENREF_8)] | 85.7%[[1-4](#_ENREF_1), [6](#_ENREF_6), [7](#_ENREF_7)] |
|  | Education | 2[[9](#_ENREF_9)] [[10](#_ENREF_10)] | 5 | 1 | 20.0% | 100.0% | 0.0% |
|  | Insurance | 3 [[1](#_ENREF_1), [6](#_ENREF_6), [11](#_ENREF_11)] | 6 | 2 | 33.3% | 50.0% [[6](#_ENREF_6)] | 50.0% [[1](#_ENREF_1)] |
|  | Smoking status | 6 [[1](#_ENREF_1), [3](#_ENREF_3), [12-15](#_ENREF_12)] | 26 | 14 | 53.8% | 85.7%[[12](#_ENREF_12)] | 14.3% [[1](#_ENREF_1), [3](#_ENREF_3)] |
|  | Alcohol use | 2 [[1](#_ENREF_1), [3](#_ENREF_3)] | 2 | 2 | 100.0% | 100.0% [[1](#_ENREF_1), [3](#_ENREF_3)] | 0.0% |
| Comorbidities | Cardiovascular disease | 2[[16](#_ENREF_16), [17](#_ENREF_17)] | 2 | 0 | 0.0% | 0.0% | 0.0% |
|  | Atrial fibrillation | 1[[7](#_ENREF_7)] | 1 | 1 | 100.0% | 100.0%[[7](#_ENREF_7)] | 0.0% |
|  | Dyslipidaemia | 3[[7](#_ENREF_7), [18](#_ENREF_18), [19](#_ENREF_19)] | 4 | 4 | 100.0% | 0.0% | 100.0%[[7](#_ENREF_7), [18](#_ENREF_18), [19](#_ENREF_19)] |
|  | CVD/ cerebrovascular disease | 1[[2](#_ENREF_2)] | 1 | 0 | 0.0% | 0.0% | 0.0% |
|  | Cerebrovascular/ stroke | 4 [[1](#_ENREF_1), [7](#_ENREF_7), [19](#_ENREF_19), [20](#_ENREF_20)] | 5 | 5 | 100.0% | 100.0% [[1](#_ENREF_1), [7](#_ENREF_7), [19](#_ENREF_19), [20](#_ENREF_20)] | 0.0% |
|  | Amputation | 2[[1](#_ENREF_1), [9](#_ENREF_9)] | 3 | 3 | 100.0% | 100.0%[[1](#_ENREF_1), [9](#_ENREF_9)] | 0.0% |
|  | Peripheral vascular disease | 5[[1-3](#_ENREF_1), [7](#_ENREF_7), [15](#_ENREF_15)] | 5 | 4 | 80.0% | 100.0% [[1-3](#_ENREF_1), [7](#_ENREF_7), [15](#_ENREF_15)] | 0.0% |
|  | Dementia | 1[[7](#_ENREF_7)] | 1 | 1 | 100.0% | 100.0%[[7](#_ENREF_7)] | 0.0% |
|  | Cognitive status/ function | 2[[21](#_ENREF_21), [22](#_ENREF_22)] | 5 | 0 | 0.0% | 0.0% | 0.0% |
|  | Memory dysfunction | 1[[22](#_ENREF_22)] | 1 | 1 | 100.0% | 100.0%[[22](#_ENREF_22)] | 0.0% |
|  | Delirium | 1[[23](#_ENREF_23)] | 1 | 1 | 100.0% | 100.0%[[23](#_ENREF_23)] | 100.0% |
|  | Depression status | 2[[14](#_ENREF_14), [21](#_ENREF_21)] | 3 | 2 | 66.7% | 100.0%[[14](#_ENREF_14), [21](#_ENREF_21)] | 0.0% |
|  | Neoplasia | 1[[20](#_ENREF_20)] | 1 | 1 | 100.0% | 100.0%[[20](#_ENREF_20)] | 0.0% |
|  | Cancer | 3 [[1](#_ENREF_1), [3](#_ENREF_3), [7](#_ENREF_7)] | 3 | 3 | 100.0% | 100.0% [[1](#_ENREF_1), [3](#_ENREF_3), [7](#_ENREF_7)] | 0.0% |
|  | Anemia | 1[[24](#_ENREF_24)] | 12 | 10 | 83.3% | 90.0%[[24](#_ENREF_24)] | 10.0%[[24](#_ENREF_24)] |
|  | Liver disease | 4[[6](#_ENREF_6), [7](#_ENREF_7), [19](#_ENREF_19), [20](#_ENREF_20)] | 7 | 6 | 85.7% | 100.0%[[6](#_ENREF_6), [7](#_ENREF_7), [19](#_ENREF_19), [20](#_ENREF_20)] | 0.0% |
|  | Lung disease/ chronic obstructive pulmonary disease (COPD)  /bronchiectasis | 5 [[1-3](#_ENREF_1), [7](#_ENREF_7), [15](#_ENREF_15)] | 6 | 5 | 83.3% | 100.0% [[1-3](#_ENREF_1), [7](#_ENREF_7), [15](#_ENREF_15)] | 0.0% |
|  | Falls/ fall injury | 1[[25](#_ENREF_25)] | 1 | 1 | 100.0% | 100.0%[[25](#_ENREF_25)] | 0.0% |
|  | Gout | 1[[26](#_ENREF_26)] | 1 | 1 | 100.0% | 0.0%[[26](#_ENREF_26)] | 100.0% |
|  | Methicillin-resistant staphylococcus aureus (MRSA) status | 1[[27](#_ENREF_27)] | 1 | 1 | 100.0% | 100.0%[[27](#_ENREF_27)] | 0.0% |
|  | Visuospatial dysfunction | 1[[22](#_ENREF_22)] | 1 | 0 | 0.0% | 0.0% | 0.0% |
|  | Systolic blood pressure (SBP)/ diastolic blood pressure (DBP) | 9[[5](#_ENREF_5), [14](#_ENREF_14), [17](#_ENREF_17), [28-33](#_ENREF_28)] | 39 | 33 | 84.6% | 48.5%[^29^](#_ENREF_29) [^30^](#_ENREF_30) ^[^[^31^](#_ENREF_31)^,^ [^32^](#_ENREF_32)^]^ | 21.2%[[5](#_ENREF_5), [14](#_ENREF_14), [32](#_ENREF_32), [33](#_ENREF_33)] |
|  | Hypertension | 9 [[1-3](#_ENREF_1), [13](#_ENREF_13), [19](#_ENREF_19), [20](#_ENREF_20), [32](#_ENREF_32), [34](#_ENREF_34), [35](#_ENREF_35)] | 25 | 18 | 72.0% | 33.3%[[32](#_ENREF_32)] | 66.7% [[1-3](#_ENREF_1), [13](#_ENREF_13), [19](#_ENREF_19), [20](#_ENREF_20), [32](#_ENREF_32), [34](#_ENREF_34), [35](#_ENREF_35)] |
| Intradialytic blood pressure | Intradialytic hypotension (edited from % with intradialytic event) | 1[[28](#_ENREF_28)] | 10 | 4 | 40.0% | 100.0%[[28](#_ENREF_28)] | 0.0% |
|  | Intradialytic hypertension | 1[[36](#_ENREF_36)] | 27 | 10 | 37.0% | 100.0%[[36](#_ENREF_36)] | 0.0% |
| Biomarkers | Apolipoprotein A-I | 1[[37](#_ENREF_37)] | 3 | 2 | 66.7% | 100.0%[[37](#_ENREF_37)] | 0.0% |
|  | B type natriuretic peptide (BNP) | 4[[30](#_ENREF_30), [33](#_ENREF_33), [38](#_ENREF_38), [39](#_ENREF_39)] | 5 | 4 | 80.0% | 100.0%[[30](#_ENREF_30), [33](#_ENREF_33), [38](#_ENREF_38), [39](#_ENREF_39)] | 0.0% |
|  | LDL / triglycerides | 6[[13](#_ENREF_13), [15](#_ENREF_15), [17](#_ENREF_17), [37](#_ENREF_37), [40](#_ENREF_40), [41](#_ENREF_41)] | 13 | 0 | 0.0% | 0.0% | 0.0% |
|  | Iron | 2[[17](#_ENREF_17), [42](#_ENREF_42)] | 2 | 1 | 50.0% | 0.0% | 100.0%[[42](#_ENREF_42)] |
|  | Transferrin saturation | 1[[43](#_ENREF_43)] | 1 | 0 | 0.0% | 0.0% | 0.0% |
|  | Erythropoietin resistance index | 1[[43](#_ENREF_43)] | 1 | 0 | 0.0% | 0.0% | 0.0% |
|  | Total iron binding capacity | 1[[44](#_ENREF_44)] | 1 | 1 | 100.0% | 100.0%[[44](#_ENREF_44)] | 0.0% |
|  | Parathyroid hormone | 5[[13](#_ENREF_13), [17](#_ENREF_17), [43](#_ENREF_43), [45](#_ENREF_45), [46](#_ENREF_46)] | 5 | 2 | 40.0% | 50.0%[[45](#_ENREF_45)] | 50.0%[[46](#_ENREF_46)] |
|  | Bilirubin | 1[[41](#_ENREF_41)] | 5 | 5 | 100.0% | 80.0%[[41](#_ENREF_41)] | 20.0%[[41](#_ENREF_41)] |
|  | Alkaline phosphatase | 1[[7](#_ENREF_7)] | 1 | 1 | 100.0% | 100.0%[[7](#_ENREF_7)] | 0.0% |
|  | Calcium-phosphorus product | 1[[42](#_ENREF_42)] | 1 | 1 | 100.0% | 100.0%[[42](#_ENREF_42)] | 0.0% |
|  | Fibroblast growth factor | 1[[47](#_ENREF_47)] | 1 | 1 | 100.0% | 100.0%[[47](#_ENREF_47)] | 0.0% |
|  | Phosphate | 3[[13](#_ENREF_13), [48](#_ENREF_48)] | 5 | 3 | 60.0% | 100.0%[[13](#_ENREF_13), [48](#_ENREF_48)] | 0.0% |
|  | Calcium | 3[[13](#_ENREF_13), [35](#_ENREF_35), [48](#_ENREF_48)] | 5 | 3 | 60.0% | 100.0%[[13](#_ENREF_13), [35](#_ENREF_35)] | 0.0% |
|  | Potassium | 3[[30](#_ENREF_30), [49](#_ENREF_49), [50](#_ENREF_50)] | 17 | 2 | 11.8% | 100.0%[[50](#_ENREF_50)] | 0.0% |
|  | Phosphorus | 2[[14](#_ENREF_14), [35](#_ENREF_35)] | 2 | 0 | 0.0% | 0.0% | 0.0% |
|  | Sodium | 4[[5](#_ENREF_5), [7](#_ENREF_7), [17](#_ENREF_17), [31](#_ENREF_31)] | 13 | 8 | 61.5% | 37.5%[[5](#_ENREF_5)] | 62.5%[[5](#_ENREF_5), [7](#_ENREF_7)] |
|  | Anion gap | 1[[51](#_ENREF_51)] | 2 | 2 | 100.0% | 100.0%[[51](#_ENREF_51)] | 0.0% |
|  | Soluble receptor for advanced glycation end products | 1[[52](#_ENREF_52)] | 1 | 0 | 0.0% | 0.0% | 0.0% |
|  | Beta 2 microglobulin | 1[[17](#_ENREF_17)] | 1 | 0 | 0.0% | 0.0% | 0.0% |
|  | Prooxidant-antioxidant balance | 1[[40](#_ENREF_40)] | 1 | 1 | 100.0% | 100.0%[[40](#_ENREF_40)] | 0.0% |
|  | Antioxidative status | 1[[40](#_ENREF_40)] | 1 | 0 | 0.0% | 0.0% | 0.0% |
|  | Paraoxonase | 2[[37](#_ENREF_37), [53](#_ENREF_53)] | 10 | 6 | 60.0% | 0.0% | 100.0%[[37](#_ENREF_37)] |
|  | Malondialdehyde | 1[[54](#_ENREF_54)] | 1 | 0 | 0.0% | 0.0% | 0.0% |
|  | Magnesium | 1 [[55](#_ENREF_55)] | 1 | 1 | 100.0% | 0.0% | 100.0% [[55](#_ENREF_55)] |
|  | Nitric oxide | 1[[42](#_ENREF_42)] | 1 | 1 | 100.0% | 100.0%[[42](#_ENREF_42)] | 0.0% |
|  | Fetuin | 1[[56](#_ENREF_56)] | 1 | 0 | 0.0% | 0.0% | 0.0% |
|  | Pentraxin 3 | 1[[53](#_ENREF_53)] | 1 | 1 | 100.0% | 100.0%[[53](#_ENREF_53)] | 0.0% |
|  | Ferritin | 4[[17](#_ENREF_17), [42](#_ENREF_42), [43](#_ENREF_43), [57](#_ENREF_57)] | 17 | 4 | 23.5% | 100.0%[[42](#_ENREF_42), [57](#_ENREF_57)] | 0.0% |
|  | Interleukin-6 | 2[[54](#_ENREF_54), [56](#_ENREF_56)] | 2 | 1 | 50.0% | 100.0%[[54](#_ENREF_54)] | 0.0% |
|  | Calciprotein particles | 1[[58](#_ENREF_58)] | 1 | 0 | 0.0% | 0.0% | 0.0% |
|  | Hepcidin | 1[[43](#_ENREF_43)] | 8 | 8 | 100.0% | 100.0% | 0.0% |
|  | Neutrophil to lymphocyte ratio | 1[[5](#_ENREF_5)] | 3 | 3 | 100.0% | 100.0%[[5](#_ENREF_5)] | 0.0% |
|  | White blood cell count | 2[[7](#_ENREF_7), [41](#_ENREF_41)] | 2 | 2 | 100.0% | 100.0%[[7](#_ENREF_7), [41](#_ENREF_41)] | 0.0% |
|  | Interferon lambda-3 (IFN-L3) | 1[[59](#_ENREF_59)] | 1 | 1 | 100.0% | 0.0% | 100.0%[[59](#_ENREF_59)] |
|  | Tumour necrosis factor alpha | 1[[53](#_ENREF_53)] | 1 | 0 | 0.0% | 0.0% | 0.0% |
|  | Fetuin-A gene | 1[[56](#_ENREF_56)] | 1 | 0 | 0.0% | 0.0% | 0.0% |
|  | HbA1c | 1[[60](#_ENREF_60)] | 9 | 3 | 33.3% | 100.0%[[60](#_ENREF_60)] | 0.0% |
|  | Hypoglycaemia | 1[[19](#_ENREF_19)] | 4 | 4 | 100.0% | 100.0%[[19](#_ENREF_19)] | 0.0% |
|  | Creatinine | 9[[5](#_ENREF_5), [13](#_ENREF_13), [15](#_ENREF_15), [17](#_ENREF_17), [30](#_ENREF_30), [43](#_ENREF_43), [47](#_ENREF_47), [61](#_ENREF_61), [62](#_ENREF_62)] | 14 | 9 | 64.3% | 0.0% | 100.0%[[5](#_ENREF_5), [13](#_ENREF_13), [15](#_ENREF_15), [30](#_ENREF_30), [47](#_ENREF_47), [62](#_ENREF_62)] |
| Cardiovascular measurements | Cardiac troponin T | 2[[40](#_ENREF_40), [54](#_ENREF_54)] | 2 | 1 | 50.0% | 100.0%[[54](#_ENREF_54)] | 0.0% |
|  | Cardio thoracic ratio | 1[[33](#_ENREF_33)] | 1 | 0 | 0.0% | 0.0% | 0.0% |
|  | Left ventricular ejection fraction | 1[[58](#_ENREF_58)] | 1 | 0 | 0.0% | 0.0% | 0.0% |
|  | Oxygen extraction rate | 1 [[63](#_ENREF_63)] | 2 | 2 | 100.0% | 50.0% [[63](#_ENREF_63)] | 50.0% [[63](#_ENREF_63)] |
|  | Proprotein convertase subtilisin | 1[[64](#_ENREF_64)] | 2 | 1 | 50.0% | 0.0% | 100.0%[[64](#_ENREF_64)] |
|  | Pulse rate | 1[[17](#_ENREF_17)] | 1 | 0 | 0.0% | 0.0% | 0.0% |
|  | Suppression of tumorigenicity | 1[[38](#_ENREF_38)] | 3 | 3 | 100.0% | 100.0%[[38](#_ENREF_38)] | 0.0% |
|  | Ankle brachial index | 1[[58](#_ENREF_58)] | 1 | 0 | 0.0% | 0.0% | 0.0% |
|  | Pulse wave velocity | 1[[65](#_ENREF_65)] | 1 | 0 | 0.0% | 0.0% | 0.0% |
| Frailty status | Frailty status | 4[[21](#_ENREF_21), [45](#_ENREF_45), [66](#_ENREF_66), [67](#_ENREF_67)] | 11 | 10 | 90.9% | 100.0%[[21](#_ENREF_21), [45](#_ENREF_45), [66](#_ENREF_66), [67](#_ENREF_67)] | 0.0% |
|  | Geriatric assessment | 1[[21](#_ENREF_21)] | 1 | 1 | 100.0% | 100.0%[[21](#_ENREF_21)] | 0.0% |
|  | Skeletal muscle mass | 1[[66](#_ENREF_66)] | 1 | 0 | 0.0% | 0.0% | 0.0% |
|  | Body cell mass index | 1[[68](#_ENREF_68)] | 1 | 1 | 100.0% | 100.0%[[68](#_ENREF_68)] | 0.0% |
|  | Dietary intake | 1[[42](#_ENREF_42)] | 1 | 1 | 100.0% | 0.0% | 100.0%[[42](#_ENREF_42)] |
|  | Malnutrition inflammation atherosclerosis | 1[[35](#_ENREF_35)] | 4 | 3 | 75.0% | 100.0%[[35](#_ENREF_35)] | 0.0% |
|  | Normalised protein catabolic rate | 1[[69](#_ENREF_69)] | 1 | 0 | 0.0% | 0.0% | 0.0% |
|  | Nutritional score | 1[[69](#_ENREF_69)] | 1 | 0 | 0.0% | 100.0%[[69](#_ENREF_69)] | 0.0% |
|  | Nutritional status | 4[[21](#_ENREF_21), [29](#_ENREF_29), [61](#_ENREF_61), [70](#_ENREF_70)] | 6 | 6 | 100.0% | 50.0%[[21](#_ENREF_21), [61](#_ENREF_61)] | 50.0%[[29](#_ENREF_29), [70](#_ENREF_70)] |
|  | Nutritional supplement use | 1[[71](#_ENREF_71)] | 1 | 1 | 100.0% | 0.0% | 100.0%[[71](#_ENREF_71)] |
|  | Salt intake | 1[[72](#_ENREF_72)] | 65 | 23 | 35.4% | 0.0% | 100.0%[[72](#_ENREF_72)] |
|  | Lean mass index | 1[[42](#_ENREF_42)] | 1 | 1 | 100.0% | 0.0% | 100.0%[[42](#_ENREF_42)] |
|  | Skinfold thickness | 1[[42](#_ENREF_42)] | 1 | 1 | 100.0% | 0.0% | 100.0%[[42](#_ENREF_42)] |
|  | Physical activity | 1 [[73](#_ENREF_73)] | 1 | 1 | 100.0% | 0.0% | 100.0% [[73](#_ENREF_73)] |
| Medications | ACEi/ARB | 1[[74](#_ENREF_74)] | 3 | 0 | 0.0% | 0.0% | 0.0% |
|  | Beta blocker | 2[[75](#_ENREF_75), [76](#_ENREF_76)] | 16 | 8 | 50.0% | 12.5%[[76](#_ENREF_76)] | 87.5%[[75](#_ENREF_75)] |
|  | Warfarin use | 1[[26](#_ENREF_26)] | 1 | 1 | 100.0% | 100.0%[[26](#_ENREF_26)] | 0.0% |
|  | Statin | 2[[77](#_ENREF_77), [78](#_ENREF_78)] | 37 | 35 | 94.6% | 0.0% | 100.0%[[77](#_ENREF_77), [78](#_ENREF_78)] |
|  | Opioid | 1[[79](#_ENREF_79)] | 6 | 5 | 83.3% | 100.0%[[79](#_ENREF_79)] | 0.0% |
|  | Vitamin D | 2[[42](#_ENREF_42), [80](#_ENREF_80)] | 7 | 7 | 100.0% | 100.0%[[42](#_ENREF_42), [80](#_ENREF_80)] | 0.0% |
|  | Vitamin D receptor agonist | 1[[34](#_ENREF_34)] | 1 | 1 | 100.0% | 0.0% | 100.0%[[34](#_ENREF_34)] |
|  | Metabolic bone management | 1[[26](#_ENREF_26)] | 1 | 0 | 0.0% | 0.0% | 0.0% |
|  | Xanthine oxidoreductase inhibitor | 1[[17](#_ENREF_17)] | 7 | 6 | 85.7% | 0.0% | 100.0%[[17](#_ENREF_17)] |
|  | Iron administration strategy | 1[[81](#_ENREF_81)] | 8 | 4 | 50.0% | 100.0%[[81](#_ENREF_81)] | 0.0% |
|  | No. of medications | 1[[26](#_ENREF_26)] | 6 | 4 | 66.7% | 100.0%[[26](#_ENREF_26)] | 0.0% |
| Treatment related indicators | Access flow | 1[[82](#_ENREF_82)] | 1 | 1 | 100.0% | 100.0%[[82](#_ENREF_82)] | 0.0% |
|  | Kt/V (dialysis adequacy) | 1[[17](#_ENREF_17)] | 1 | 0 | 0.0% | 0.0% | 0.0% |
|  | Vascular access type | 9[[2](#_ENREF_2), [3](#_ENREF_3), [5](#_ENREF_5), [8](#_ENREF_8), [27](#_ENREF_27), [83-85](#_ENREF_83)] | 23 | 17 | 77.3% | 100.0%[[2](#_ENREF_2), [3](#_ENREF_3), [5](#_ENREF_5), [27](#_ENREF_27), [84](#_ENREF_84)] | 0.0% |
|  | Haemodialysis shunt | 0 | 0 | 0 | . | . | . |
|  | Central venous catheter infection | 1[[86](#_ENREF_86)] | 1 | 1 | 100.0% | 100.0%[[86](#_ENREF_86)] | 0.0% |
|  | Dialysis duration per session | 1[[87](#_ENREF_87)] | 1 | 1 | 100.0% | 0.0% | 100.0%[[87](#_ENREF_87)] |
|  | Dialysis vintage | 6[[13](#_ENREF_13), [15](#_ENREF_15), [17](#_ENREF_17), [27](#_ENREF_27), [33](#_ENREF_33), [58](#_ENREF_58)] | 7 | 2 | 28.6% | 100.0%[[15](#_ENREF_15), [27](#_ENREF_27)] | 0.0% |
|  | Dialyzer membrane | 2[[88](#_ENREF_88), [89](#_ENREF_89)] | 24 | 10 | 41.7% | 30.0%[[88](#_ENREF_88), [89](#_ENREF_89)] | 70.0%[[88](#_ENREF_88), [89](#_ENREF_89)] |
|  | Pressor approaches | 1[[31](#_ENREF_31)] | 12 | 8 | 66.7% | 100.0%[[31](#_ENREF_31)] | 0.0% |
|  | Predialysis fluid status | 1[[90](#_ENREF_90)] | 23 | 21 | 91.3% | 95.2%[[90](#_ENREF_90)] | 4.8%[[90](#_ENREF_90)] |
|  | Over hydration | 1[[5](#_ENREF_5)] | 3 | 3 | 100.0% | 100.0%[[5](#_ENREF_5)] | 0.0% |
|  | Dialysis provider type | 3 [[91-93](#_ENREF_91)] | 29 | 24 | 81.5% | 0.0% | 100.0%[[91-93](#_ENREF_91)] |
|  | Region of dialysis facility | 1[[6](#_ENREF_6)] | 2 | 2 | 100.0% | 50.0%[[6](#_ENREF_6)] | 50.0%[[6](#_ENREF_6)] |
|  | Inpatient dialysis therapy initiation | 1[[94](#_ENREF_94)] | 1 | 1 | 100.0% | 100.0%[[94](#_ENREF_94)] | 0.0% |
|  | Vasopressor use | 1[[31](#_ENREF_31)] | 2 | 2 | 100.0% | 100.0%[[31](#_ENREF_31)] | 0.0% |
|  | High or low volume predilution online hemodiafiltration | 1[[95](#_ENREF_95)] | 1 | 1 | 100.0% | 0.0% | 100.0%[[95](#_ENREF_95)] |
|  | Suboptimal initiation of dialysis | 1[[34](#_ENREF_34)] | 8 | 5 | 62.5% | 100.0%[[34](#_ENREF_34)] | 0.0% |
|  | Urgent/ conventional start of dialysis | 1[[96](#_ENREF_96)] | 1 | 0 | 0.0% | 0.0% | 0.0% |
|  | Non emergent start dialysis vs emergent start dialysis | 3 [[18](#_ENREF_18), [97](#_ENREF_97), [98](#_ENREF_98)] | 4 | 3 | 75.0% | 100.0% [[18](#_ENREF_18), [97](#_ENREF_97), [98](#_ENREF_98)] | 0.0% |
|  | Peritoneal dialysis versus haemodialysis | 7 [[1-3](#_ENREF_1), [9](#_ENREF_9), [19](#_ENREF_19), [45](#_ENREF_45), [99](#_ENREF_99)] | 10 | 10 | 100.0% | 90.0% [[1-3](#_ENREF_1), [9](#_ENREF_9), [19](#_ENREF_19), [45](#_ENREF_45)] | 10.0% [[99](#_ENREF_99)] |
| Renal related parameters | Blood urea nitrogen (BUN) | 3[[7](#_ENREF_7), [13](#_ENREF_13), [17](#_ENREF_17)] | 3 | 1 | 33.3% | 100.0%[[7](#_ENREF_7)] | 0.0% |
|  | estimated glomerular filtration rate (eGFR) | 3[[7](#_ENREF_7), [16](#_ENREF_16), [86](#_ENREF_86)] | 3 | 2 | 66.7% | 100.0%[[7](#_ENREF_7), [86](#_ENREF_86)] | 0.0% |
|  | Uremic score | 1[[100](#_ENREF_100)] | 1 | 1 | 100.0% | 100%%[[100](#_ENREF_100)] | 0.0% |
|  | Uric acid | 2[[27](#_ENREF_27), [35](#_ENREF_35)] | 2 | 1 | 50.0% | 0.0% | 100.0%[[35](#_ENREF_35)] |
|  | Duration of ESRD | 1[[1](#_ENREF_1)] | 1 | 1 | 100.0% | 0.0% | 100.0%[[1](#_ENREF_1)] |
| Health status | Health literacy score | 1 [[10](#_ENREF_10)] | 1 | 1 | 100.0% | 0.0% | 100.0% [[10](#_ENREF_10)] |
|  | Health related QOL | 2[[101](#_ENREF_101), [102](#_ENREF_102)] | 40 | 14 | 35.0% | 78.6%[[101](#_ENREF_101), [102](#_ENREF_102)] | 21.4%[[102](#_ENREF_102)] |
|  | No prior medical evaluation | 1[[86](#_ENREF_86)] | 1 | 0 | 0.0% | 0.0% | 0.0% |
|  | Body pain | 1[[101](#_ENREF_101)] | 4 | 0 | 0.0% | 0.0% | 0.0% |
|  | Charlson comorbidity index (CCI) | 2 [[10](#_ENREF_10), [27](#_ENREF_27)] | 4 | 2 | 50.0% | 100.0%[[27](#_ENREF_27)] | 0.0% |
| Cause of ESRD | Cause of ESRD | 8[[1-3](#_ENREF_1), [7](#_ENREF_7), [16](#_ENREF_16), [33](#_ENREF_33), [59](#_ENREF_59), [86](#_ENREF_86)] | 33 | 24 | 72.7% | 66.7%[[1-3](#_ENREF_1), [7](#_ENREF_7), [59](#_ENREF_59)] | 33.3%[[1](#_ENREF_1)] [[2](#_ENREF_2), [3](#_ENREF_3), [86](#_ENREF_86)] |
| Access to healthcare information | Access to transplant information | 2[[2](#_ENREF_2), [3](#_ENREF_3)] | 2 | 2 | 100.0% | 0.0% | 100.0%[[2](#_ENREF_2), [3](#_ENREF_3)] |
|  | Pre-ESRD care | 3[[1-3](#_ENREF_1)] | 9 | 8 | 88.9% | 25.0%[[1](#_ENREF_1), [3](#_ENREF_3)] | 75.0%[[1-3](#_ENREF_1)] |
|  | Setting of pre-ESRD care | 1[[103](#_ENREF_103)] | 2 | 2 | 100.0%[[103](#_ENREF_103)] | 100.0% | 0.0% |
| Poor outcome indicator | Readmission to hospital | 2[[104](#_ENREF_104), [105](#_ENREF_105)] | 10 | 10 | 100.0% | 100.0%[[104](#_ENREF_104), [105](#_ENREF_105)] | 0.0% |
|  | History of hospitalisation | 4[[2](#_ENREF_2), [3](#_ENREF_3), [105](#_ENREF_105), [106](#_ENREF_106)] | 13 | 13 | 100.0% | 100.0%[[2](#_ENREF_2), [3](#_ENREF_3), [105](#_ENREF_105), [106](#_ENREF_106)] | 0.0% |
|  | Risk score | 2[[30](#_ENREF_30), [65](#_ENREF_65)] | 6 | 6 | 100.0% | 100.0%[[30](#_ENREF_30), [65](#_ENREF_65)] | 0.0% |

**Supplementary figure 1: Domains of risk factors assessed for mortality < 1 year**

**Supplementary figure 2: Domains of risk factors assessed for mortality within 1-3 years**

**References**

1. Shah S, Leonard AC, Meganathan K, Christianson AL, Thakar CV: **Temporal Trends in Incident Mortality in Dialysis Patients: Focus on Sex and Racial Disparities**. (1421-9670 (Electronic)).

2. Shah SA-O, Leonard AC, Thakar CV: **Functional status, pre-dialysis health and clinical outcomes among elderly dialysis patients**. (1471-2369 (Electronic)).

3. Shah SA-O, Meganathan K, Christianson AL, Leonard AC, Thakar CV: **Pre-dialysis acute care hospitalizations and clinical outcomes in dialysis patients**. (1932-6203 (Electronic)).

4. Shah S, Leonard AC, Meganathan K, Christianson AL, Thakar CV: **Gender and Racial Disparities in Initial Hemodialysis Access and Outcomes in Incident End-Stage Renal Disease Patients**. (1421-9670 (Electronic)).

5. Ye X, Kooman JP, van der Sande FM, Canaud B, Stuard S, Etter M, Xu X, Marelli C, Guinsburg A, Power A *et al*: **Increased Mortality Associated with Higher Pre-Dialysis Serum Sodium Variability: Results of the International MONitoring Dialysis Outcome Initiative**. (1421-9670 (Electronic)).

6. Deshpande R, Stepanova M, Golabi PA-O, Brown K, Younossi ZA-OX: **Prevalence, mortality and healthcare utilization among Medicare beneficiaries with Hepatitis C in Haemodialysis units**. (1365-2893 (Electronic)).

7. Obi Y, Nguyen DV, Zhou H, Soohoo M, Zhang L, Chen Y, Streja E, Sim JJ, Molnar MZ, Rhee CM *et al*: **Development and Validation of Prediction Scores for Early Mortality at Transition to Dialysis**. (1942-5546 (Electronic)).

8. Pike SL, Farber A, Arinze N, Levin S, Cheng TW, Jones DW, Tan TW, Malas M, Rybin D, Siracuse JJ: **Patients with lower extremity dialysis access have poor primary patency and survival**. (1097-6809 (Electronic)).

9. Bujang MA, Kuan PX, Sapri FE, Liu WJ, Musa R: **Risk Factors for 3-Year-Mortality and a Tool to Screen Patient in Dialysis Population**. (0971-4065 (Print)).

10. Griva KA-O, Yoong RKL, Nandakumar M, Rajeswari M, Khoo EYH, Lee VYW, Kang AWC, Osborne RA-O, Brini S, Newman SP: **Associations between health literacy and health care utilization and mortality in patients with coexisting diabetes and end-stage renal disease: A prospective cohort study**. (2044-8287 (Electronic)).

11. Nee R, Fisher E Fau - Yuan CM, Yuan Cm Fau - Agodoa LY, Agodoa Ly Fau - Abbott KC, Abbott KC: **Pre-End-Stage Renal Disease Care and Early Survival among Incident Dialysis Patients in the US Military Health System**. (1421-9670 (Electronic)).

12. Li NC, Thadhani RI, Reviriego-Mendoza M, Larkin JW, Maddux FW, Ofsthun NJ: **Association of Smoking Status With Mortality and Hospitalization in Hemodialysis Patients**. (1523-6838 (Electronic)).

13. Sueta DA-O, Tabata N, Tanaka M, Hanatani S, Arima Y, Sakamoto K, Yamamoto E, Izumiya Y, Kaikita K, Arizono K *et al*: **Associations between corrected serum calcium and phosphorus levels and outcome in dialysis patients in the Kumamoto Prefecture**. (1542-4758 (Electronic)).

14. Saglimbene V, Palmer S, Scardapane M, Craig JC, Ruospo M, Natale P, Gargano L, Leal M, Bednarek-Skublewska A, Dulawa J *et al*: **Depression and all-cause and cardiovascular mortality in patients on haemodialysis: a multinational cohort study**. (1460-2385 (Electronic)).

15. Park JM, Lee JA-O, Jang HM, Park Y, Kim YS, Kang SW, Yang CW, Kim NH, Kwon E, Kim HJ *et al*: **Survival in patients on hemodialysis: Effect of gender according to body mass index and creatinine**. (1932-6203 (Electronic)).

16. Jiang J, Wang LH, Fei YY, Zhou XW, Peng L, Lan L, Ren W: **Serum Albumin at Start of Peritoneal Dialysis Predicts Long-Term Outcomes in Anhui Han Patients on Continuous Ambulatory Peritoneal Dialysis: A Retrospective Cohort Study**. (2296-9381 (Print)).

17. Ishii T, Taguri M, Tamura K, Oyama K: **Evaluation of the Effectiveness of Xanthine Oxidoreductase Inhibitors on Haemodialysis Patients using a Marginal Structural Model**. (2045-2322 (Electronic)).

18. Li WY, Wang YC, Hwang SJ, Lin SH, Wu KD, Chen YM: **Comparison of outcomes between emergent-start and planned-start peritoneal dialysis in incident ESRD patients: a prospective observational study**. (1471-2369 (Electronic)).

19. Hsiao CC, Tu HT, Lin CH, Chen KH, Yeh YH, See LC: **Temporal Trends of Severe Hypoglycemia and Subsequent Mortality in Patients with Advanced Diabetic Kidney Diseases Transitioning to Dialysis. LID - 10.3390/jcm8040420 [doi] LID - 420**. (2077-0383 (Print)).

20. Artru FA-O, Louvet A, Glowacki F, Bellati S, Frimat M, Gomis S, Castel H, Barthelon J, Lassailly G, Dharancy S *et al*: **The prognostic impact of cirrhosis on patients receiving maintenance haemodialysis**. (1365-2036 (Electronic)).

21. van Loon IN, Goto NA, Boereboom FTJ, Bots ML, Hoogeveen EK, Gamadia L, van Bommel EFH, van de Ven PJG, Douma CE, Vincent HH *et al*: **Geriatric Assessment and the Relation with Mortality and Hospitalizations in Older Patients Starting Dialysis**. (2235-3186 (Electronic)).

22. Zhang YH, Yang ZK, Wang JW, Xiong ZY, Liao JL, Hao L, Liu GL, Ren YP, Wang Q, Duan LP *et al*: **Cognitive Changes in Peritoneal Dialysis Patients: A Multicenter Prospective Cohort Study**. (1523-6838 (Electronic)).

23. Arai Y, Shioji S, Tanaka H, Kondo I, Sakamoto E, Suzuki M, Katagiri D, Tada M, Hinoshita F: **Delirium is independently associated with early mortality in elderly patients starting hemodialysis**. (1437-7799 (Electronic)).

24. Wetmore JA-O, Li S, Yan H, Xu H, Peng Y, Sinsakul MV, Liu J, Gilbertson DT: **Predialysis anemia management and outcomes following dialysis initiation: A retrospective cohort analysis**. (1932-6203 (Electronic)).

25. Bowling CB, Hall RK, Khakharia A, Franch HA, Plantinga LC: **Serious Fall Injury History and Adverse Health Outcomes After Initiating Hemodialysis Among Older U.S. Adults**. (1758-535X (Electronic)).

26. Samaranayaka S, Walker RA-O, Samaranayaka A, Derrett S, Schollum JWB: **Medication Exposure and Health Outcomes in Older Patients with End-Stage Kidney Disease: A Prospective Study Undertaken in New Zealand**. (1179-1969 (Electronic)).

27. Price AM, Sarween N, Gupta I, Baharani J: **Risk factors and short-term outcomes for methicillin-resistant Staphylococcus aureus and methicillin-sensitive Staphylococcus aureus colonization among hemodialysis patients**. (1319-2442 (Print)).

28. Chou JA, Streja E, Nguyen DV, Rhee CM, Obi Y, Inrig JK, Amin A, Kovesdy CP, Sim JJ, Kalantar-Zadeh K: **Intradialytic hypotension, blood pressure changes and mortality risk in incident hemodialysis patients**. (1460-2385 (Electronic)).

29. Shimamura YA-O, Maeda T Fau - Abe K, Abe K Fau - Takizawa H, Takizawa H: **Association of blood pressure with mortality in hemodialysis patients with a tunneled cuffed catheter: A single-center observational study**. (1536-5964 (Electronic)).

30. Komaru Y, Yoshida T, Hamasaki Y, Nangaku M, Doi K: **Hierarchical Clustering Analysis for Predicting 1-Year Mortality After Starting Hemodialysis**. (2468-0249 (Electronic)).

31. Kanda E, Tsuruta Y, Kikuchi K, Masakane I: **Use of vasopressor for dialysis-related hypotension is a risk factor for death in hemodialysis patients: Nationwide cohort study**. (2045-2322 (Electronic)).

32. Dekker M, Konings C, Canaud B, Carioni P, Guinsburg A, Madero M, van der Net J, Raimann J, van der Sande F, Stuard S *et al*: **Pre-dialysis fluid status, pre-dialysis systolic blood pressure and outcome in prevalent haemodialysis patients: results of an international cohort study on behalf of the MONDO initiative**. (1460-2385 (Electronic)).

33. Kawagoe C, Sato Y, Toida T, Nakagawa H, Yamashita Y, Fukuda A, Iwatsubo S, Fujimoto S: **N-terminal-pro-B-type-natriuretic peptide associated with 2-year mortality from both cardiovascular and non-cardiovascular origins in prevalent chronic hemodialysis patients**. (1525-6049 (Electronic)).

34. Kanno A, Nakayama M, Sanada S, Sato M, Sato T, Taguma Y: **Suboptimal initiation predicts short-term prognosis and vulnerability among very elderly patients who start haemodialysis**. (1440-1797 (Electronic)).

35. Sueta D, Hokimoto S, Sakamoto K, Akasaka T, Tabata N, Kaikita K, Honda O, Naruse M, Ogawa H: **Validation of the high mortality rate of Malnutrition-Inflammation-Atherosclerosis syndrome: -Community-based observational study**. (1874-1754 (Electronic)).

36. Assimon MM, Wang L, Flythe JE: **Intradialytic Hypertension Frequency and Short-Term Clinical Outcomes Among Individuals Receiving Maintenance Hemodialysis**. (1941-7225 (Electronic)).

37. Suematsu Y, Goto M, Park C, Nunes ACF, Jing W, Streja E, Rhee CM, Cruz S, Kashyap ML, Vaziri ND *et al*: **Association of Serum Paraoxonase/Arylesterase Activity With All-Cause Mortality in Maintenance Hemodialysis Patients**. (1945-7197 (Electronic)).

38. Homsak E, Ekart R: **ST2 as a novel prognostic marker in end-stage renal disease patients on hemodiafiltration**. (1873-3492 (Electronic)).

39. Arrigo M, Von Moos S, Gerritsen K, Sadoune M, Tangvoraphonkchai K, Davenport A, Mebazaa A, Segerer S, Cippà PE: **Soluble CD146 and B-type natriuretic peptide dissect overhydration into functional components of prognostic relevance in haemodialysis patients**. (1460-2385 (Electronic)).

40. Antunovic T, Stefanovic A, Gligorovic Barhanovic N, Miljkovic M, Radunovic D, Ivanisevic J, Prelevic V, Bulatovic N, Ratkovic M, Stojanov M: **Prooxidant-antioxidant balance, hsTnI and hsCRP: mortality prediction in haemodialysis patients, two-year follow-up**. (1525-6049 (Electronic)).

41. Yang TL, Lin YC, Huang CY, Chen HH, Wu MS: **Total Bilirubin in Prognosis for Mortality in End-Stage Renal Disease Patients on Peritoneal Dialysis Therapy. LID - 10.1161/JAHA.117.007507 [doi] LID - e007507**. (2047-9980 (Electronic)).

42. Balbino KA-O, Juvanhol LL, Epif√¢nio APS, Marota LD, Bressan J, Hermsdorff HHM: **Dietary intake as a predictor for all-cause mortality in hemodialysis subjects (NUGE-HD study)**. (1932-6203 (Electronic)).

43. Wang LSL-XZYLNDH-X: **Effect of Serum Hepcidin on Predicting Mortality in Hemodialysis Patients: A Prospective Cohort Study**. *Iranian Red Cresent Medical Journal* 2019, **21**(3):7.

44. Bai KJ, Huang KC, Lee CH, Tang CH, Yu MC, Sue YM: **Effect of pulmonary tuberculosis on clinical outcomes of long-term dialysis patients: Pre- and post-DOTS implementation in Taiwan**. (1440-1843 (Electronic)).

45. Lee SY, Yang DH, Hwang E, Kang SH, Park SH, Kim TW, Lee DH, Park K, Kim JC: **The Prevalence, Association, and Clinical Outcomes of Frailty in Maintenance Dialysis Patients**. (1532-8503 (Electronic)).

46. Villa-Bellosta R, Rodriguez-Osorio L, Mas S, Abadi Y, Rubert M, de la Piedra C, Gracia-Iguacel C, Mahillo I, Ortiz A, Egido J *et al*: **A decrease in intact parathyroid hormone (iPTH) levels is associated with higher mortality in prevalent hemodialysis patients**. (1932-6203 (Electronic)).

47. Nielsen TL, Plesner LL, Warming PE, Mortensen OH, Iversen KK, Heaf JG: **FGF23 in hemodialysis patients is associated with left ventricular hypertrophy and reduced ejection fraction**. (2013-2514 (Electronic)).

48. Lee JE, Lim JH, Jang HM, Kim YS, Kang SW, Yang CW, Kim NH, Kwon E, Kim HJ, Park JM *et al*: **Low serum phosphate as an independent predictor of increased infection-related mortality in dialysis patients: A prospective multicenter cohort study**. (1932-6203 (Electronic)).

49. Brunelli SM, Spiegel DM, Du Mond C, Oestreicher N, Winkelmayer WC, Kovesdy CP: **Serum-to-dialysate potassium gradient and its association with short-term outcomes in hemodialysis patients**. (1460-2385 (Electronic)).

50. Brunelli SM, Du Mond C, Oestreicher N, Rakov V, Spiegel DM: **Serum Potassium and Short-term Clinical Outcomes Among Hemodialysis Patients: Impact of the Long Interdialytic Interval**. (1523-6838 (Electronic)).

51. Arai Y, Tanaka H, Shioji S, Sakamoto E, Kondo I, Suzuki M, Katagiri D, Tada M, Hinoshita F: **Anion gap predicts early mortality after starting hemodialysis in the elderly**. (1437-7799 (Electronic)).

52. Dozio EA-O, Ambrogi FA-OX, de Cal MA-O, Vianello EA-OX, Ronco C, Corsi Romanelli MA-O: **Role of the Soluble Receptor for Advanced Glycation End Products (sRAGE) as a Prognostic Factor for Mortality in Hemodialysis and Peritoneal Dialysis Patients**. (1466-1861 (Electronic)).

53. Valente MA-O, Rocha S, Coimbra SA-O, Catarino C, Rocha-Pereira P, Bronze-da-Rocha EA-O, Oliveira JG, Madureira J, Fernandes JC, do Sameiro-Faria MA-O *et al*: **Long Pentraxin 3 as a Broader Biomarker for Multiple Risk Factors in End-Stage Renal Disease: Association with All-Cause Mortality**. (1466-1861 (Electronic)).

54. Wang Z, Yu C, Li XH, Deng BQ: **The prognostic value of oxidative stress and inflammation in Chinese hemodialysis patients**. (1525-6049 (Electronic)).

55. Lu C, Wang Y, Wang D, Nie L, Zhang Y, Lei Q, Xiong J, Zhao J: **Hypomagnesemia and Short-Term Mortality in Elderly Maintenance Hemodialysis Patients**. (2296-9381 (Print)).

56. Muzasti RA, Suhardjono D, Purwanto B, Sembiring RJ: **Fetuin-A Thr256Ser gene polymorphism as a mortality predictor in patients with chronic kidney disease on maintenance haemodialysis in Medan, Indonesia**. (1840-2445 (Electronic)).

57. Karaboyas A, Morgenstern H, Pisoni RL, Zee J, Vanholder R, Jacobson SH, Inaba M, Loram LC, Port FK, Robinson BM: **Association between serum ferritin and mortality: findings from the USA, Japan and European Dialysis Outcomes and Practice Patterns Study**. (1460-2385 (Electronic)).

58. Gatate Y, Nakano S, Mizuno Y, Muramatsu T, Senbonmatsu T, Nishimura S, Kono R, Kaneko K, Miura Y, Kuro OM: **Mid-term predictive value of calciprotein particles in maintenance hemodialysis patients based on a gel-filtration assay**. (1879-1484 (Electronic)).

59. Grzegorzewska AE, Swiderska MK, Warchol W: **Interferon-Œª3 as a Predictor of Survival in Hemodialysis Patients**. (1875-5666 (Electronic)).

60. Hoshino J, Hamano T, Abe M, Hasegawa T, Wada A, Ubara Y, Takaichi K, Inaba M, Nakai S, Masakane I: **Glycated albumin versus hemoglobin A1c and mortality in diabetic hemodialysis patients: a cohort study**. (1460-2385 (Electronic)).

61. Borges MC, Vogt BP, Martin LC, Caramori JC: **Malnutrition Inflammation Score cut-off predicting mortality in maintenance hemodialysis patients**. (2405-4577 (Electronic)).

62. Zhou SJ, Cong YK, Han QF, Tang W, Wang T: **Peritoneal dialysis outcomes in patients with nephrotic syndrome: a propensity score-matched cohort study**. (1525-6049 (Electronic)).

63. Rotondi S, Tartaglione L, Muci ML, Farcomeni AA-O, Pasquali M, Mazzaferro SA-O: **Oxygen Extraction Ratio (OER) as a Measurement of Hemodialysis (HD) Induced Tissue Hypoxia: A Pilot Study**. (2045-2322 (Electronic)).

64. Strålberg T, Nordenskjöld A, Cao Y, Kublickiene K, Nilsson EA-O: **Proprotein convertase subtilisin/kexin type 9 and mortality in patients starting hemodialysis**. (1365-2362 (Electronic)).

65. Tripepi G, Agharazii M, Pannier B, D'Arrigo G, Mallamaci F, Zoccali C, London G: **Pulse Wave Velocity and Prognosis in End-Stage Kidney Disease**. (1524-4563 (Electronic)).

66. Kamijo Y, Kanda E, Ishibashi Y, Yoshida M: **Sarcopenia and Frailty in PD: Impact on Mortality, Malnutrition, and Inflammation**. (1718-4304 (Electronic)).

67. Lorenz EC, Cosio FG, Bernard SL, Bogard SD, Bjerke BR, Geissler EN, Hanna SW, Kremers WK, Cheng Y, Stegall MD *et al*: **The Relationship Between Frailty and Decreased Physical Performance With Death on the Kidney Transplant Waiting List**. (2164-6708 (Electronic)).

68. Oliveira T, Garagarza C, Valente A, Caetano C: **Low body cell mass index in hemodialysis patients: Association with clinical parameters and survival**. (1542-4758 (Electronic)).

69. Chen HS, Cheng CT, Hou CC, Liou HH, Chang CT, Lin CJ, Wu TK, Chen CH, Lim PS: **A Practical Standardized Composite Nutrition Score Based on Lean Tissue Index: Application in Nutrition Screening and Prediction of Outcome in Hemodialysis Population**. (1532-8503 (Electronic)).

70. Ye X, Dekker MJE, Maddux FW, Kotanko P, Konings C, Raimann JG, van der Sande FM, Usvyat LA, Kooman JP, Thijssen S: **Dynamics of Nutritional Competence in the Last Year Before Death in a Large Cohort of US Hemodialysis Patients**. (1532-8503 (Electronic)).

71. Benner D, Brunelli SM, Brosch B, Wheeler J, Nissenson AR: **Effects of Oral Nutritional Supplements on Mortality, Missed Dialysis Treatments, and Nutritional Markers in Hemodialysis Patients**. (1532-8503 (Electronic)).

72. Ikenoue T, Koike K, Fukuma S, Ogata S, Iseki K, Fukuhara S: **Salt Intake and All-Cause Mortality in Hemodialysis Patients**. (1421-9670 (Electronic)).

73. Zhang L, Luo H, Kang G, Wang W, Hu Y: **The association between physical activity and mortality among patients undergoing maintenance hemodialysis. LID - 10.1111/ijn.12505 [doi]**. (1440-172X (Electronic)).

74. Shen JI, Saxena AB, Montez-Rath ME, Leng L, Chang TI, Winkelmayer WC: **Comparative effectiveness of angiotensin receptor blockers vs. angiotensin-converting enzyme inhibitors on cardiovascular outcomes in patients initiating peritoneal dialysis**. (1724-6059 (Electronic)).

75. Zhou H, Sim JJ, Shi J, Shaw SF, Lee MS, Neyer JR, Kovesdy CP, Kalantar-Zadeh K, Jacobsen SJ: **Œ≤-Blocker Use and Risk of Mortality in Heart Failure Patients Initiating Maintenance Dialysis**. (1523-6838 (Electronic)).

76. Assimon MM, Brookhart MA, Fine JP, Heiss G, Layton JB, Flythe JE: **A Comparative Study of Carvedilol Versus Metoprolol Initiation and 1-Year Mortality Among Individuals Receiving Maintenance Hemodialysis**. (1523-6838 (Electronic)).

77. Soohoo M, Moradi H, Obi Y, Rhee CM, Gosmanova EO, Molnar MZ, Kashyap ML, Gillen DL, Kovesdy CP, Kalantar-Zadeh K *et al*: **Statin Therapy Before Transition to End-Stage Renal Disease With Posttransition Outcomes**. (2047-9980 (Electronic)).

78. Streja E, Gosmanova EO, Molnar MZ, Soohoo M, Moradi H, Potukuchi PK, Kalantar-Zadeh K, Kovesdy CP: **Association of Continuation of Statin Therapy Initiated Before Transition to Chronic Dialysis Therapy With Mortality After Dialysis Initiation**. (2574-3805 (Electronic)).

79. Waddy SP, Becerra AZ, Ward JB, Chan KE, Fwu CW, Eggers PW, Abbott KC, Kimmel PL: **Concomitant Use of Gabapentinoids with Opioids Is Associated with Increased Mortality and Morbidity among Dialysis Patients**. (1421-9670 (Electronic)).

80. Villa-Bellosta R, Mahillo-Fern√°ndez I, Ort√≠z A, Gonz√°lez-Parra E: **Questioning the Safety of Calcidiol in Hemodialysis Patients. LID - 10.3390/nu11050959 [doi] LID - 959**. (2072-6643 (Electronic)).

81. Li XA-O, Cole SR, Kshirsagar AV, Fine JP, Stürmer T, Brookhart MA: **Safety of Dynamic Intravenous Iron Administration Strategies in Hemodialysis Patients**. (1555-905X (Electronic)).

82. Wu CK, Wu CL, Lin CH, Leu JG, Kor CT, Tarng DC: **Association of vascular access flow with short-term and long-term mortality in chronic haemodialysis patients: a retrospective cohort study**. (2044-6055 (Electronic)).

83. Soleymanian T, Kokabeh Z, Ramaghi R, Mahjoub A, Argani H: **Clinical outcomes and quality of life in hemodialysis diabetic patients versus non-diabetics**. (2251-8363 (Print)).

84. Saleh T, Sumida K Fau - Molnar MZ, Molnar Mz Fau - Potukuchi PK, Potukuchi Pk Fau - Thomas F, Thomas F Fau - Lu JL, Lu Jl Fau - Gyamlani GG, Gyamlani Gg Fau - Streja E, Streja E Fau - Kalantar-Zadeh K, Kalantar-Zadeh K Fau - Kovesdy CP, Kovesdy CP: **Effect of Age on the Association of Vascular Access Type with Mortality in a Cohort of Incident End-Stage Renal Disease Patients**. (2235-3186 (Electronic)).

85. Ryan TJ, Farber A, Cheng TW, Raulli SJ, Sather K, Dicken QG, Levin SR, Zhang Y, Siracuse JJ: **Factors associated with a tunneled dialysis catheter in place at initial arteriovenous access creation**. (1097-6809 (Electronic)).

86. G√≥mez de la Torre-Del Carpio A, Bocanegra-Jes√∫s A, Guinetti-Ortiz K, Mayta-Trist√°n P, Valdivia-Vega R: **Early mortality in patients with chronic kidney disease who started emergency haemodialysis in a Peruvian population: Incidence and risk factors**. (2013-2514 (Electronic)).

87. Swaminathan S, Mor V, Mehrotra R, Trivedi AN: **Initial Session Duration and Mortality Among Incident¬†Hemodialysis Patients**. (1523-6838 (Electronic)).

88. Abe M, Hamano T Fau - Wada A, Wada A Fau - Nakai S, Nakai S Fau - Masakane I, Masakane I: **High-Performance Membrane Dialyzers and Mortality in Hemodialysis Patients: A 2-Year Cohort Study from the Annual Survey of the Japanese Renal Data Registry**. (1421-9670 (Electronic)).

89. Abe MA-O, Hamano T, Wada A, Nakai S, Masakane I: **Effect of dialyzer membrane materials on survival in chronic hemodialysis patients: Results from the annual survey of the Japanese Nationwide Dialysis Registry**. (1932-6203 (Electronic)).

90. Dekker MJ, Marcelli D, Canaud BJ, Carioni P, Wang Y, Grassmann A, Konings CJ, Kotanko P, Leunissen KM, Levin NW *et al*: **Impact of fluid status and inflammation and their interaction on survival: a study in an international hemodialysis patient cohort**. (1523-1755 (Electronic)).

91. Streja EA-O, Kovesdy CA-O, Soohoo M, Obi YA-O, Rhee CA-O, Park C, Chen JLT, Nakata T, Nguyen DV, Amin AN *et al*: **Dialysis Provider and Outcomes among United States Veterans Who Transition to Dialysis**. (1555-905X (Electronic)).

92. Lin MA-O, Cheng LJ, Chiu YW, Hsieh HM, Wu PH, Lin YT, Wang SL, Jian FX, Hsu CC, Yang SA *et al*: **Effect of national pre-ESRD care program on expenditures and mortality in incident dialysis patients: A population-based study**. (1932-6203 (Electronic)).

93. Ajmal F, Probst JC, Brooks JM, Hardin JW, Qureshi Z, Jafar TH: **Freestanding Dialysis Facility Quality Incentive Program Scores and Mortality Among Incident Dialysis Patients in the United States**. (1523-6838 (Electronic)).

94. Arif FM, Sumida K Fau - Molnar MZ, Molnar Mz Fau - Potukuchi PK, Potukuchi Pk Fau - Lu JL, Lu Jl Fau - Hassan F, Hassan F Fau - Thomas F, Thomas F Fau - Siddiqui OA, Siddiqui Oa Fau - Gyamlani GG, Gyamlani Gg Fau - Kalantar-Zadeh K, Kalantar-Zadeh K Fau - Kovesdy CP *et al*: **Early Mortality Associated with Inpatient versus Outpatient Hemodialysis Initiation in a Large Cohort of US Veterans with Incident End-Stage Renal Disease**. (2235-3186 (Electronic)).

95. Kikuchi K, Hamano T, Wada A, Nakai S, Masakane I: **Predilution online hemodiafiltration is associated¬†with improved survival compared with¬†hemodialysis**. (1523-1755 (Electronic)).

96. Phang CC, Foo MWY, Johnson DW, Wu SY, Hao Y, Jayaballa M, Koniman R, Chan CM, Oei EL, Chong TT *et al*: **Comparison of outcomes of urgent-start and conventional-start peritoneal dialysis: a single-centre experience**. (1573-2584 (Electronic)).

97. Nguyen OK, Vazquez MA, Charles L, Berger JR, Quiñones H, Fuquay R, Sanders JM, Kapinos KA, Halm EA, Makam AN: **Association of Scheduled vs Emergency-Only Dialysis With Health Outcomes and Costs in Undocumented Immigrants With End-stage Renal Disease**. (2168-6114 (Electronic)).

98. van Loon IN, Goto NA, Boereboom FTJ, Verhaar MC, Bots ML, Hamaker ME: **Quality of life after the initiation of dialysis or maximal conservative management in elderly patients: a longitudinal analysis of the Geriatric assessment in OLder patients starting Dialysis (GOLD) study**. (1471-2369 (Electronic)).

99. Wong B, Ravani P, Oliver MJ, Holroyd-Leduc J, Venturato L, Garg AX, Quinn RR: **Comparison of Patient Survival Between Hemodialysis and Peritoneal Dialysis Among Patients Eligible for Both Modalities**. (1523-6838 (Electronic)).

100. Arai Y, Shioji S, Tanaka H, Katagiri D, Hinoshita F: **A Novel Uremic Score Reflecting Accumulation of Specific Uremic Toxins More Precisely Predicts One-Year Mortality after Hemodialysis Commencement: A Retrospective Cohort Study. LID - 10.3390/toxins12100634 [doi] LID - 634**. (2072-6651 (Electronic)).

101. van Loon IN, Bots ML, Boereboom FTJ, Grooteman MPC, Blankestijn PJ, van den Dorpel MA, Nub√© MJ, Ter Wee PM, Verhaar MC, Hamaker ME: **Quality of life as indicator of poor outcome in hemodialysis: relation with mortality in different age groups**. (1471-2369 (Electronic)).

102. Perl J, Karaboyas A, Morgenstern H, Sen A, Rayner HC, Vanholder RC, Combe C, Hasegawa T, Finkelstein FO, Lopes AA *et al*: **Association between changes in quality of life and mortality in hemodialysis patients: results from the DOPPS**. (1460-2385 (Electronic)).

103. Kurella Tamura M, Thomas IC, Montez-Rath ME, Kapphahn K, Desai M, Gale RC, Asch SM: **Dialysis Initiation and Mortality Among Older Veterans With Kidney Failure Treated in Medicare vs the Department of Veterans Affairs**. (2168-6114 (Electronic)).

104. Plantinga LC, King L, Patzer RE, Lea JP, Burkart JM, Hockenberry JM, Jaar BG: **Early hospital readmission among hemodialysis patients in the United States is associated with subsequent mortality**. (1523-1755 (Electronic)).

105. Ross KA-O, Jaar BG, Lea JP, Masud T, Patzer RE, Plantinga LC: **Long-term outcomes among Medicare patients readmitted in the first year of hemodialysis: a retrospective cohort study**. (1471-2369 (Electronic)).

106. Kimmel PL, Fwu CW, Abbott KC, Moxey-Mims MM, Mendley S, Norton JA-O, Eggers PW: **Psychiatric Illness and Mortality in Hospitalized ESKD Dialysis Patients**. (1555-905X (Electronic)).
